# Associations of soundscape naturalness and natural-element complexity with short-term psychophysiological restoration in campus green spaces: a virtual reality study

**DOI:** 10.3389/fpsyg.2026.1947314

**Published:** 2026-09-23

**Authors:** Jiaqi Zhang, Xiaoyang Guo

**Affiliations:** School of Architecture and Urban Planning, Suzhou University of Science and Technology, Suzhou, China

**Keywords:** campus green infrastructure, natural-element complexity, psychophysiological restoration, restorative environment, soundscape naturalness, sustainable built environment, virtual reality

## Abstract

Sustainable campus development increasingly requires outdoor environments that support both environmental quality and student wellbeing. Yet campus green spaces are commonly evaluated through visible greenness, while the combined roles of natural visual structure and soundscape naturalness remain unclear. This study integrated natural-element fractal dimension (NEFD) and the Green Soundscape Index (GSI) to assess short-term psychophysiological responses to campus green spaces. Seventy-three audiovisual scenes were collected from 10 campuses in Suzhou, China, and nine representative NEFD–GSI conditions were selected for a repeated-measures virtual reality experiment. Forty students were recruited, with valid data from 35 participants. Visual-only and audiovisual exposures were evaluated using subjective ratings, eye tracking, electrodermal activity, and cardiovascular indicators. Across the nine sampled scene conditions, response differences were most consistently aligned with the GSI dimension; for pupil diameter, the omnibus GSI-related contrast was substantial, *F*(2, 578) = 241.15, *q* < 0.001, partial *η*^2^ = 0.455. NEFD-related differences showed a non-monotonic and soundscape-dependent pattern, with medium-NEFD conditions showing favourable tendencies under medium and high GSI; under medium GSI, the low–medium NEFD pupil-diameter contrast was 0.452 (95% CI [0.352, 0.552], Tukey-adjusted *p* < 0.001). Electrodermal effects were more selective, whereas most cardiovascular indicators showed limited differentiation. These findings describe sample-specific response differences among nine Suzhou campus scene conditions and do not establish generalisable effects attributable independently to NEFD or GSI; the identified numerical ranges should not be interpreted as universal design thresholds.

## 1 Introduction

Sustainable urban development is increasingly concerned not only with ecological provision and environmental performance, but also with the capacity of everyday built environments to support health, wellbeing, and psychological recovery. This issue is particularly relevant to university campuses, where students are repeatedly exposed to academic, social, and residential stressors. Mental-health problems among university students represent a substantial public-health burden. A systematic review and meta-analysis of 89 studies involving approximately 130,090 undergraduate students reported a weighted mean prevalence of 39.65% for non-specific anxiety (95% CI: 35.72–43.58%) (Ahmed et al., 2023). More recently, a nationwide survey of 41,620 students from 106 Chinese universities reported detection rates of 9.8% for depression, 15.5% for anxiety, and 6.5% for depression–anxiety comorbidity (Han et al., 2025). Together, these findings highlight both the broader scale and the specific relevance of student mental-health challenges in the Chinese university context, reinforcing the importance of viewing campuses as everyday health-supportive environments alongside their educational function. Within this setting, campus blue and green spaces represent a particularly accessible form of health-supportive infrastructure because they are embedded in students’ daily routines and can provide opportunities for psychological recovery. Campus blue and green spaces may contribute to restoration and self-rated health (Liu Q. et al., 2022; Aghabozorgi et al., 2024), while broader evidence links nature exposure with stress reduction, attention recovery, physical activity, social connection, and other biopsychosocial pathways (World Health Organization, 2016; World Health Organization, 2017; Hartig et al., 2014; Bratman et al., 2019; Jimenez et al., 2021).

Within this broader health-supportive context, university campuses constitute a particularly relevant setting because they combine dense learning, residential, social, and circulation environments with frequently encountered outdoor green spaces (Huang et al., 2024; Sun et al., 2024). Unlike large destination parks, campus blue–green spaces are embedded in daily routines, encountered repeatedly, and generally accessible without additional travel. They can therefore provide brief opportunities for recovery between academic and social demands. Recent evidence increasingly suggests that such benefits can emerge over relatively short exposure periods and need not follow a simple linear dose–response pattern. Yao et al. (2024), for example, showed that physiological benefits of greenspace exposure can emerge within minutes and proposed distinct efficiency and benefit thresholds, while also emphasising that these responses vary with contextual conditions. Related planning research has further operationalised a 15-min effective-exposure criterion within an integrated accessibility–exposure framework (Wang X. et al., 2026; Wang Y. et al., 2026). Recent campus-based evidence further indicates that restorative outcomes depend not merely on the presence or quantity of blue–green space, but on the characteristics of the exposure actually experienced by students (Dai et al., 2026), using psychological and neurophysiological assessments across nine university campuses, found that three-dimensional eye-level visual metrics explained immediate psychological restoration more effectively than conventional two-dimensional green-space measures and exhibited nonlinear threshold patterns. Together with recent evidence that campus restorative outcomes depend on users’ perception and experience of environmental conditions, including audiovisual characteristics (Xu et al., 2024), these findings shift the research question from whether campus greenery is beneficial to which characteristics of brief environmental exposure are most relevant to restoration.

This shift from green-space quantity to restorative performance also raises an important measurement issue. Restorative-environment and window-view studies commonly foreground visual attributes such as greenery, vegetation proportion, openness, complexity, and overall view quality (Herzog et al., 2003; Shibata et al., 2024; Sun et al., 2024; Song et al., 2025). Conventional vegetation-coverage and visible-green indicators primarily quantify how much greenery is present or visible, while measures of openness or enclosure describe spatial exposure and containment. Recent campus research further demonstrates the value of moving beyond plan-based green quantity toward eye-level visual exposure (Dai et al., 2026). However, the amount of visible greenery remains conceptually distinct from the geometric organisation of the natural elements that constitute a scene. In particular, the structural organisation of vegetation edges, branching forms, water boundaries, and terrain contours has received less systematic quantification (Laumann et al., 2001; Han, 2003; Hagerhall et al., 2004; Stamps, 2004; Liu et al., 2021; Robles et al., 2021). In the present study, natural-element fractal dimension (NEFD) is therefore defined specifically as the contour-based structural complexity of segmented natural elements. It is intended to represent natural-element boundary complexity rather than visible green quantity, whole-scene visual complexity, or spatial openness. This distinction is important because recent work has shown that visual exposure quality cannot be fully inferred from conventional green-space quantity alone.

Visual structure, however, represents only one component of how campus green spaces are experienced (Li and Lau, 2020). In everyday settings, visually similar green spaces may differ substantially in traffic intrusion, water sounds, birdsong, human activity, conversation, or mechanical noise. Soundscape research has long demonstrated that acoustic environments cannot be reduced to noise level alone; they are perceptual environments shaped by source composition, appraisal, and context (Schafer, 1977; Alvarsson et al., 2010; Axelsson et al., 2010; Kang and Zhang, 2010; Abbott et al., 2016; Krzywicka and Byrka, 2017; Ratcliffe, 2021; Uebel et al., 2021; Liu F. et al., 2022). Natural sounds and lower anthropogenic dominance may reduce vigilance, improve affect, support stress recovery, and facilitate attention restoration (Alvarsson et al., 2010; Axelsson et al., 2010; Kang and Zhang, 2010; Abbott et al., 2016; Krzywicka and Byrka, 2017; Ratcliffe, 2021; Uebel et al., 2021; Liu F. et al., 2022; Bai and Zhang, 2024; Fan and Baharum, 2024; Gilmour et al., 2024). Recent VR studies further demonstrate that visual and acoustic characteristics jointly shape short-term stress restoration and perceived restorative potential (Jeon et al., 2023; Kawai et al., 2024; Kawai et al., 2025). To quantify acoustic source composition, the present study uses the Green Soundscape Index (GSI), which represents the relative dominance of model-recognised natural sound components over anthropogenic components (Kogan et al., 2018). GSI is therefore interpreted as a source-composition indicator rather than as sound-pressure level, the literal physical proportion of all natural sounds, or a complete measure of perceived soundscape quality. Pairing NEFD and GSI thus provides a common visual–acoustic parameter space for comparing structured campus environmental conditions.

These measurable visual and acoustic characteristics can be integrated within a broader exposure-based account of restoration. Exposure Ecology conceptualises health outcomes as downstream consequences of the pattern, process, and characteristics of ecological exposure, shifting attention from the mere presence of green space to the environmental attributes actually encountered by individuals. Recent exposure-oriented research similarly emphasises that environmental characteristics and human experience should be connected within the same exposure–health pathway rather than treated as separate spatial and health processes (Wang X. et al., 2026; Wang Y. et al., 2026). Wang et al. explicitly distinguish environmental attributes from the way people actually encounter and use green spaces, highlighting exposure as the link between environmental provision and subsequent health benefits. Recent pathway-based research provides complementary evidence that ecological characteristics can influence short-term physiological health through multiple mechanisms. Yang et al. (2026), for example, distinguished direct perceptual and aesthetic pathways from indirect microenvironmental and biochemical pathways in linking urban plant diversity with physiological responses. Within this broader exposure–health chain, NEFD and GSI can therefore be understood as two measurable sensory characteristics of audiovisual environmental exposure rather than as isolated design features.

Restorative-environment theories provide a more specific account of how these exposure characteristics may translate into short-term human responses. Attention Restoration Theory proposes that environments capable of engaging attention without excessive directed effort can facilitate recovery of attentional resources (Kaplan and Kaplan, 1989; Kaplan, 1995; Kaplan and Berman, 2010; Berman et al., 2008; Berman et al., 2012). Related perceptual-processing perspectives similarly suggest that coherent environmental configurations that can be processed relatively fluently may reduce unnecessary cognitive demand, whereas excessive complexity or conflicting sensory information may increase processing requirements. Natural complexity should therefore not automatically be assumed to produce monotonically increasing restorative benefit. Relatively simple environments may provide limited perceptual richness, whereas highly complex configurations may impose greater processing demands; intermediate levels may therefore provide a more favourable balance between perceptual richness and processing effort. Research on fractal and biodiversity-related visual structure is consistent with the possibility that natural complexity influences psychological responses without implying that greater complexity is always more restorative (Hagerhall et al., 2004; Stevens, 2018; Robles et al., 2021). More broadly, greenspace dose–response research has documented improvement followed by plateauing or decline, as well as U-shaped and inverted-U-shaped relationships, indicating that greater exposure does not necessarily yield proportionally greater health benefit (Yao et al., 2024). Campus-based research similarly reports nonlinear and threshold-dependent relationships between visual nature exposure and restorative outcomes (Dai et al., 2026). Acoustic context may further modify these relationships: anthropogenic sound dominance may increase vigilance or interfere with restorative processing, whereas greater dominance of natural sounds may provide a more supportive sensory context. The restorative relevance of natural visual complexity may therefore depend on the soundscape in which it is experienced.

Restoration is accordingly treated in the present study as a multisystem process rather than as a single subjective judgement or a single physiological mechanism. Systematic and empirical work on VR physiology indicates that different psychophysiological indicators vary in sensitivity and temporal response (Halbig and Latoschik, 2021; Sánchez-Reolid et al., 2022; Ferreira et al., 2024; Gorin et al., 2024; Yu et al., 2024). Eye-tracking studies show that gaze and pupil measures can reveal rapid changes in visual processing and cognitive effort (Liu and Zhou, 2025; Liu et al., 2025), while restorative soundscape research demonstrates the value of combining perceptual and physiological evidence (Long et al., 2023). Pupil and electrodermal measures provide complementary information on processing demand, emotional elicitation, and sympathetic arousal (Boucsein, 2012; Duchowski, 2017; van der Wel and van Steenbergen, 2018; Amin and Faghih, 2022; Polo et al., 2022; Jayawardena et al., 2025), while cardiovascular indicators provide additional evidence of autonomic regulation. In the present study, short-term psychophysiological restoration is therefore operationally defined as a multisystem pattern of responses during brief environmental exposure, encompassing subjective appraisal, pupil- and gaze-related processing measures, electrodermal arousal, and cardiovascular regulation. These response domains are treated as complementary rather than as interchangeable measures of a single latent restorative mechanism.

Taken together, the literature reveals three related gaps. First, campus green-space assessment still tends to emphasise green quantity, visual exposure, or individual sensory attributes, while the contour-based structural complexity of natural elements and acoustic source composition are rarely integrated within a common quantitative framework. Second, limited evidence explains whether combinations of measurable visual and acoustic characteristics are associated with coherent or context-dependent short-term responses across subjective, cognitive, and autonomic systems. Third, experimental findings are not always translated into environmental variables that can support design diagnosis, retrofit prioritisation, and post-occupancy evaluation (Li and Sullivan, 2016; Stoltz et al., 2016; Lai et al., 2020). Addressing these gaps requires an indicator-based approach that connects measurable environmental exposure characteristics with multisystem human-response evidence while maintaining explicit limits on causal and threshold interpretation.

To address these gaps, this study developed a VR-based visual–acoustic assessment framework for restorative campus green spaces. Using immersive VR together with eye-tracking, electrodermal, cardiovascular, and subjective measures, the study examined whether systematically selected NEFD–GSI scene conditions were associated with distinguishable short-term psychophysiological response patterns. The primary objective was to evaluate whether response patterns varied systematically along the GSI and NEFD dimensions and whether NEFD-related responses differed across soundscape contexts. Because each NEFD–GSI combination was represented by one real campus scene, the study was designed to characterise associations across structured audiovisual scene conditions rather than to isolate causal effects of NEFD or GSI independently from other scene-level attributes. Accordingly, the framework is intended as an indicator-based associative assessment approach rather than as a general predictive model or a causal test of isolated NEFD and GSI effects.

Based on restorative-environment theory and recent evidence that nature–health relationships may exhibit nonlinear and threshold-dependent patterns (Berto, 2005; Berman et al., 2008; Yao et al., 2024; He et al., 2024; Dai et al., 2026), three primary hypotheses were proposed:

*H1*. Higher GSI will be associated with more favourable short-term restorative responses, reflected in lower pupil-related processing demand, reduced electrodermal arousal, and more positive subjective evaluations.

*H2*. NEFD will show a non-monotonic association with short-term restorative responses, such that intermediate levels of natural-element structural complexity may be associated with more favourable responses than relatively low or high levels.

*H3*. The association between NEFD and short-term restorative responses will vary across GSI levels, with the inverted-U-shaped NEFD pattern expected to become more apparent under conditions with greater relative dominance of model-recognised natural sound component.

Exploratory response-surface modelling was additionally used to visualise response tendencies within the sampled NEFD–GSI parameter range and to support cautious design translation. Any favourable parameter regions identified from these analyses were interpreted as sample-specific empirical patterns requiring further validation rather than as universal design thresholds.

The study makes three connected contributions. First, it distinguishes natural-element structural complexity from conventional greenness and openness measures and integrates it with acoustic source composition within a common NEFD–GSI environmental representation. Second, it provides multisystem human-response evidence on comparatively consistent GSI-related response patterns and context-dependent NEFD-related patterns across the selected scene conditions. Third, it translates these response patterns into an exploratory indicator-based framework for campus green-space performance assessment, renewal diagnosis, retrofit prioritisation, and post-occupancy evaluation.

## 2 Materials and methods

The methodological design followed the logic of translating real campus green-space conditions into measurable audiovisual indicators and then linking these indicators with short-term psychophysiological responses. The study comprised four connected stages: multi-campus audiovisual scene collection, NEFD–GSI environmental quantification and validation, representative-scene selection and immersive VR exposure, and multisystem human-response analysis. Mixed-effects models provided the primary participant-level analytical approach, whereas response-surface modelling was used as an exploratory tool for design-oriented interpretation.

### 2.1 Study context, campus selection, and audiovisual scene collection

The study selected 10 campuses from nine representative universities in Suzhou, China, covering historical, modern planned, and international/open campus forms. Suzhou was selected because it represents a high-density educational and urban setting in eastern China, where campus green spaces are closely embedded within everyday learning, residential, and circulation environments. Its humid subtropical urban context provides a useful basis for developing an audiovisual assessment logic for campus green spaces in comparable climatic and urban conditions, while also providing a reference for future cross-climatic validation. The sampling strategy was designed to provide multi-campus evidence within a single urban context rather than treating one university as an isolated case. Campus categories were defined descriptively according to campus development history, spatial organisation, and degree of openness: historical campuses generally contained older spatial fabrics and more compact landscape settings; modern planned campuses were characterised by planned layouts and more standardised green-space systems; and international/open campuses showed stronger interfaces with surrounding urban spaces and more open spatial organisation. These categories were used as descriptive sampling strata to broaden the range of campus environments rather than as formal analytical predictors or universally applicable campus typologies. The locations and categories of the selected campuses are shown in Figure 1, and the corresponding campus codes and numbers of collected audiovisual scenes are listed in Table 1.

**Figure 1:**
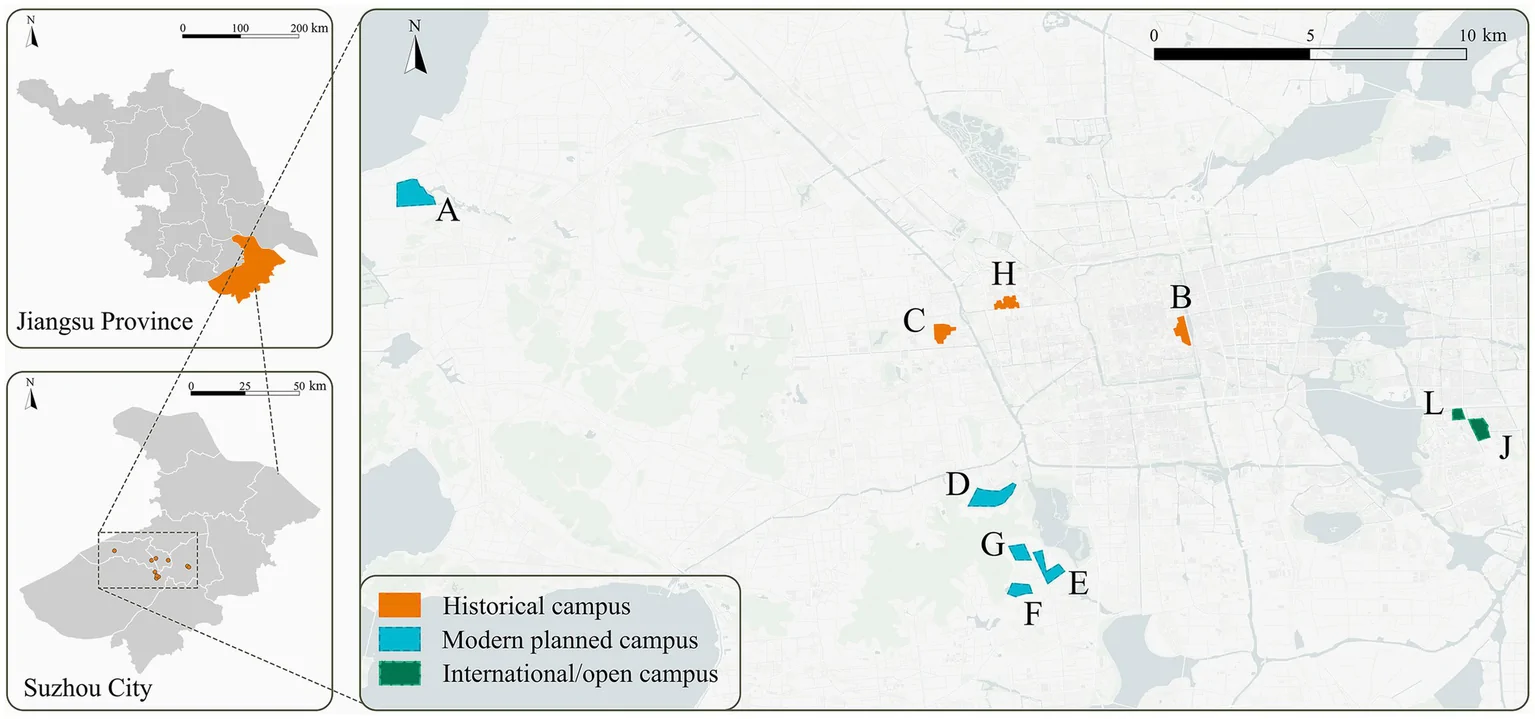
Locations and categories of the selected university campuses in Suzhou, China.

**Table 1:** Selected campuses and number of collected audiovisual scenes.

Campus code	Campus	Campus category	No. of scenes
A	Nanjing University, Suzhou Campus	Modern planned campus	7
B	Suzhou University (Tianci Zhuang Campus)	Historical campus	10
C	Suzhou University of Science and Technology (Jiangfeng Campus)	Historical campus	7
D	Suzhou University of Science and Technology (Shihu Campus)	Modern planned campus	9
E	Suzhou Polytechnic University	Modern planned campus	6
F	Suzhou City University	Modern planned campus	5
G	Suzhou Art and Design Technology Institute	Historical campus	8
H	Suzhou Polytechnic Institute of Agriculture	Historical campus	7
I	Xi’an Jiaotong-Liverpool University	International/open campus	9
J	Renmin University of China, Suzhou Campus	International/open campus	5
Total	–	–	73

Scene selection followed three principles. First, the sampled points had to be everyday accessible green spaces frequently encountered by campus users, including courtyards, shaded walkways, waterside areas, residential green spaces, and outdoor study or rest areas. Second, the scenes were selected to cover different combinations of natural visual structure and sound-source composition rather than only visually attractive or highly vegetated settings. Third, temporary disturbances and atypical activity conditions were avoided so that the audiovisual materials represented relatively stable campus environmental conditions. Field collection was conducted in October, during the early-to-mid autumn period in Suzhou, and mainly between 2:00 p.m. and 4:00 p.m. All materials were collected on weekdays under clear weather and relatively stable lighting conditions, while class-transition periods, temporary construction, unusual events, and other obvious disturbances were avoided.

Panoramic images were captured using a Canon EOS R5 camera mounted on a tripod at approximately 1.6 m above the ground, corresponding to the approximate eye height of a standing observer. For each scene, at least 20 source photographs were acquired using exposure bracketing to preserve detail across areas with different luminance levels. The images were subsequently merged and stitched in Adobe Photoshop to generate 360° equirectangular panoramas with a resolution of 16,000 × 8,000 pixels. Exposure blending, white-balance harmonisation, and seam correction were applied during post-processing to reduce visible tonal and stitching discontinuities while preserving the original spatial structure and natural colour appearance. Synchronous field recordings were obtained using a Zoom F6 recorder and a RODE NTG3 microphone positioned at the same height and oriented consistently with the corresponding panorama. The camera and microphone locations were kept spatially aligned so that each panoramic image and sound recording represented the same observation point and environmental condition.

For each scene, a 4-min field recording was obtained, from which a representative 3-min segment with stable acoustic conditions and no obvious transient noise was selected. The same 3-min segment was used for both GSI calculation and VR presentation, thereby maintaining consistency between the quantified acoustic indicator and the experimental stimulus. Audio clips were converted to WAV format and processed at 48 kHz/24-bit. Playback level was standardised across stimuli using the same digital normalisation procedure. All audio clips were presented through the same VR headset headphones using a fixed system-volume setting throughout the experiment. Because the study focused primarily on sound-source composition rather than overall sound intensity, all audio stimuli were presented at a controlled and consistent playback level.

### 2.2 Quantification of NEFD and GSI

To operationalise the visual–acoustic assessment framework proposed in the Introduction, the collected campus scenes were converted into two environmental indicators: NEFD for natural-element structural complexity and GSI for the relative dominance of model-recognised natural sound components.

Natural-element fractal dimension (NEFD) quantified the contour-based geometric complexity of natural visual components rather than the fractal dimension of the entire scene. Each panorama was converted into eight perspective projections at 45° intervals, producing 584 perspective images from the 73 scenes. Each perspective projection used a horizontal field of view of 80° and an output resolution of 1,024 × 1,024 pixels. Semantic segmentation was performed using Mask2Former trained on the Mapillary Vistas dataset (Cheng et al., 2022; Neuhold et al., 2017). Natural elements, including vegetation, trees, grass, plants, flowers, terrain, and water, were retained, whereas built structures, sky, roads, people, vehicles, and other non-natural components were excluded.

The retained natural elements were converted into binary masks. Edge extraction used a 5 × 5 Gaussian filter and the Canny detector with lower and upper thresholds of 50 and 150, respectively (Canny, 1986). The resulting edge map was multiplied by the natural-element mask so that only edge pixels located within segmented natural elements were retained. NEFD was calculated using the standard box-counting method (Foroutan-Pour et al., 1999), as shown in Equation 1:

$$\text{NEFD}=\frac{d\;\log\;N(s)}{d\;\log\;(1/s)}$$ (1)

where *N*(*s*) denotes the number of boxes containing at least one natural-element edge pixel at grid size s. The resulting NEFD value represents the contour-based fractal complexity of segmented natural landscape elements.

Acoustic source composition was quantified using the Green Soundscape Index (GSI), which represents the relative dominance of model-recognised natural sound components over anthropogenic components within each audio stimulus (Kogan et al., 2018). The 3-min audio segment presented in VR was also used for GSI calculation. Audio clips were converted to mono signals and resampled to 16 kHz before model inference. Sound-source recognition was performed using YAMNet, a MobileNet-based audio classification model trained on AudioSet (Google, n.d.; Howard et al., 2017; Gemmeke et al., 2017).

Bird, Chirp, Animal, and related biophonic labels were grouped as bird/animal sounds. Wind, Rustle, Nature, Rain, Water, and related geophonic or natural ambient labels were grouped as wind/rain/nature sounds. These categories formed the natural sound component. Speech, Crowd, Human voice, Music, Traffic, mechanical sounds, and other human-produced labels were classified as anthropogenic sound components.

To retain weak background natural sounds, frame-level prediction scores were accumulated rather than classified only by maximum probability. Scores below 0.0001 were set to zero before accumulation. The cumulative scores of natural and anthropogenic sound classes were denoted as Snatural and Santhropogenic, respectively. GSI was calculated as shown in Equation 2:

$$\mathrm{GSI}=\frac{S_{\text{natura}1}+\varepsilon }{S_{\text{natura}1}+S_{\text{anthropogenic}}+2\varepsilon }$$ (2)

where *ε* = 10–6 was used to avoid zero denominators and reduce extreme boundary values. GSI ranges from 0 to 1, with higher values indicating greater relative dominance of model-recognised natural sound components. Accordingly, the indicator was interpreted as a relative source-composition measure rather than a direct measure of sound-pressure level or a complete representation of perceived soundscape quality. The workflow for deriving NEFD and GSI is shown in Figure 2.

**Figure 2:**
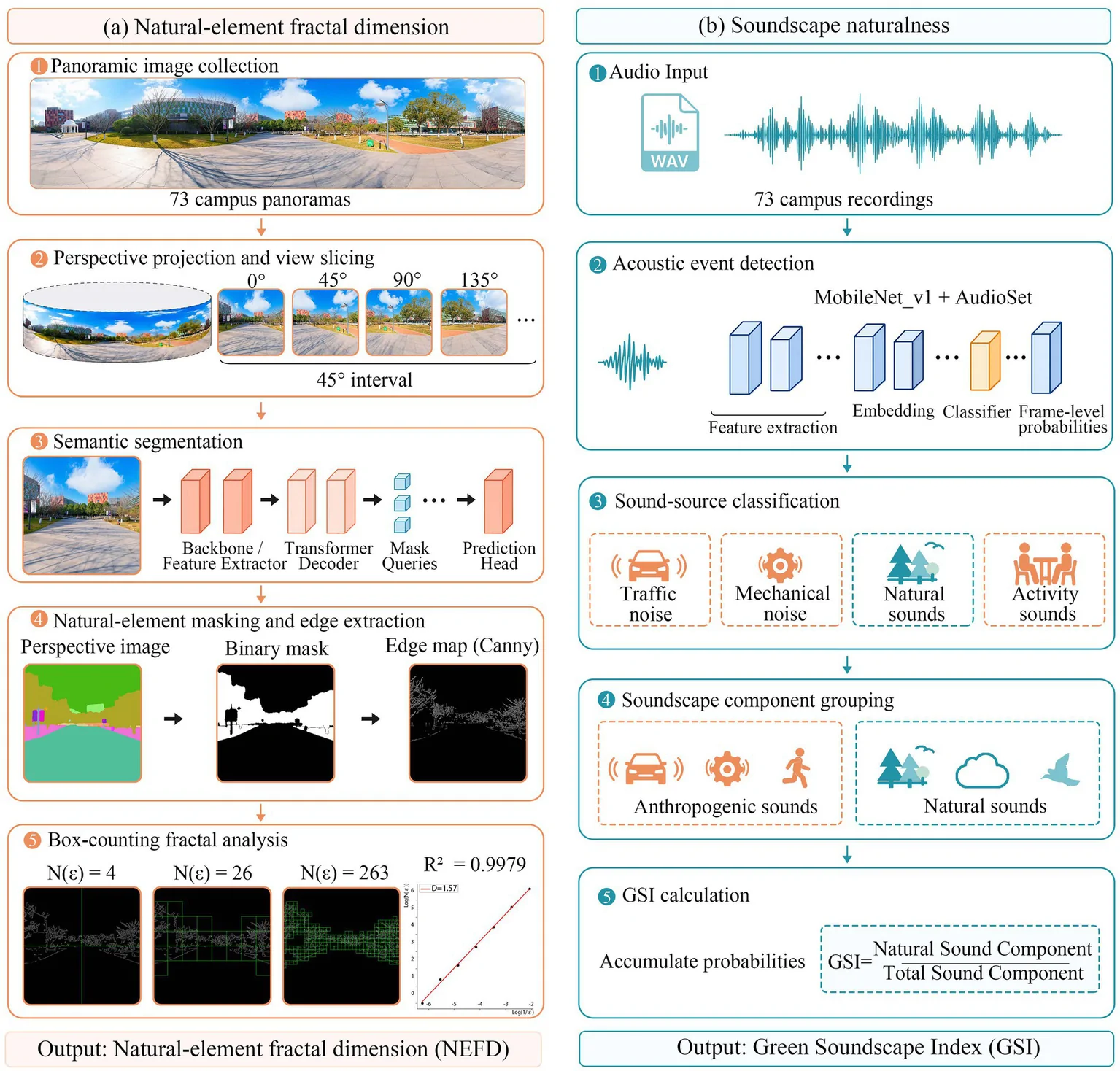
Workflow for quantifying **(a)** natural-element fractal dimension (NEFD) and **(b)** Green Soundscape Index (GSI).

### 2.3 Manual validation procedure for audiovisual indicators

Because both NEFD and GSI depended on automated image segmentation and sound-source recognition, a manual validation procedure was conducted to assess whether these indicators were reliable enough for relative comparison among the experimental scenes. For visual validation, 80 perspective images were selected from the 584 generated perspective images, including images from all nine experimental scenes and a random subset of non-experimental candidate scenes. The natural-element masks produced by Mask2Former were manually checked and corrected where necessary to assess whether vegetation, terrain, and water were retained while sky, buildings, roads, and people were excluded. A trained rater checked the visual validation subset, and a second researcher reviewed it; disagreements were resolved through discussion. Intersection over Union (IoU) and Dice coefficients were calculated to quantify agreement between the automated and manually checked masks. NEFD was then recalculated from the manually checked masks using the same box-counting procedure, and its agreement with the automatically derived NEFD was evaluated using Spearman’s rank correlation. The manually checked NEFD values were also classified using the same low–medium–high procedure, allowing weighted kappa to be calculated between automated and manually checked classifications.

For acoustic validation, the nine experimental audio clips were divided into 10-s segments, yielding 162 audio segments for manual sound-source labelling and naturalness rating. Each segment was labelled according to its dominant perceived sound-source category: natural-dominant, anthropogenic-dominant, mixed, or unclear. Two trained raters independently labelled the segments, and disagreements were resolved through discussion to generate the reference labels. Cohen’s kappa was used to quantify agreement between human-labelled and model-derived dominant sound categories, while Spearman’s rank correlation was used to examine the association between manual naturalness ratings and segment-level GSI values.

### 2.4 Selection of representative audiovisual scenes

After NEFD and GSI values were obtained for the 73 scenes, a two-dimensional audiovisual parameter space was constructed. NEFD represented the contour-based structural complexity of segmented natural elements, and GSI represented the relative dominance of model-recognised natural sound components. To avoid manually defined thresholds, K-means clustering was applied separately to NEFD and GSI values (MacQueen, 1967). Each parameter was divided into three clusters corresponding to low, medium, and high levels. Each scene was then assigned to one NEFD level and one GSI level, producing a 3 × 3 NEFD–GSI matrix. For each matrix cell, the centroid of the NEFD–GSI combination was calculated, and the scene closest to that centroid was selected as the representative audiovisual condition. This procedure produced nine representative scenes for the VR experiment. Their distribution in the NEFD–GSI parameter space is shown in Figure 3, and their environmental characteristics are listed in Table 2.

**Figure 3:**
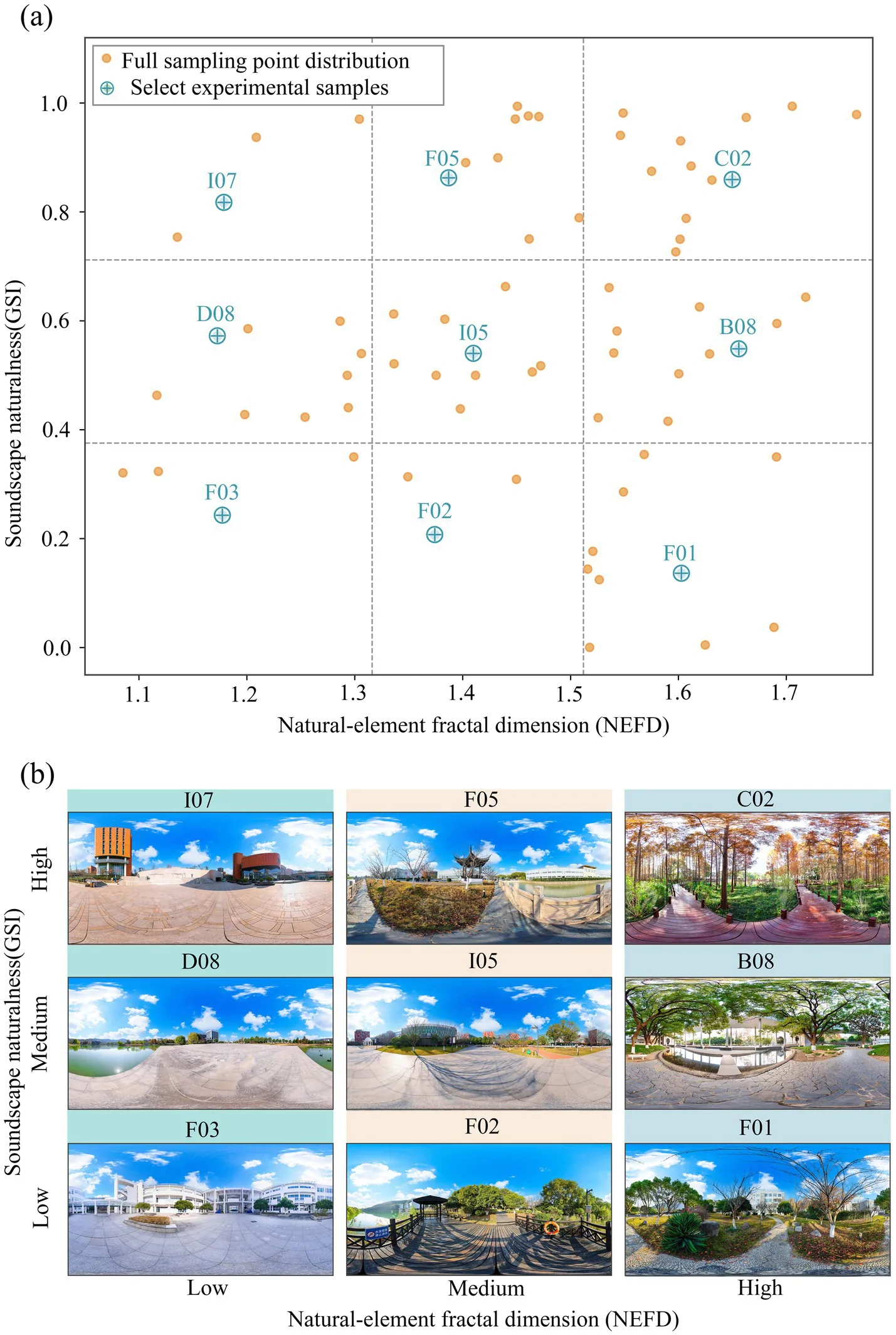
Distribution of campus scenes in the NEFD–GSI parameter space and representative panoramic images. **(a)** Distribution of the 73 sampled campus scenes and nine selected representative scenes. **(b)** Representative scenes selected from different NEFD–GSI combinations.

**Table 2:** Environmental characteristics of the nine representative scenes.

Scene ID	GSI level	GSI value	NEFD level	NEFD value	Soundscape composition (%)	Main natural visual elements (%)
F03	Low	0.2431	Low	1.1773	Natural 24.31%; Anthropogenic 75.69%	Vegetation 6.89%; Terrain 0.20%
F02	Low	0.2075	Medium	1.3741	Natural 20.75%; Anthropogenic 79.25%	Vegetation 28.80%; Terrain 2.44%; Water 2.31%
F01	Low	0.2365	High	1.6027	Natural 23.65%; Anthropogenic 76.35%	Vegetation 56.42%; Terrain 26.35%
D08	Medium	0.5325	Low	1.1645	Natural 53.25%; Anthropogenic 46.75%	Terrain 28.94%; Water 15.74%; Vegetation 5.91%
I05	Medium	0.5403	Medium	1.4099	Natural 54.03%; Anthropogenic 45.97%	Vegetation 21.33%; Terrain 6.74%
B08	Medium	0.5886	High	1.659	Natural 58.86%; Anthropogenic 41.14%	Vegetation 38.61%; Water 12.31%; Terrain 4.79%
I07	High	0.7875	Low	1.1787	Natural 78.75%; Anthropogenic 21.25%	Vegetation 5.48%; Terrain 0.50%; Water 0.16%
F05	High	0.8326	Medium	1.387	Natural 83.26%; Anthropogenic 16.74%	Vegetation 9.31%; Terrain 0.95%; Water 0.48%
C02	High	0.8594	High	1.6498	Natural 85.94%; Anthropogenic 14.06%	Vegetation 85.88%; Terrain 1.29%; Water 0.32%

The representative-scene strategy balanced ecological validity and experimental control. Real campus panoramas and field recordings preserved the spatial and sensory character of actual green spaces, while the 3 × 3 selection procedure ensured coverage of low, medium, and high combinations of the two focal indicators. However, because each NEFD–GSI cell was represented by only one real scene, scene identity could not be separated completely from the corresponding indicator combination. Vegetation proportion, water visibility, openness, enclosure, built interfaces, and other scene-level attributes may therefore covary with NEFD and GSI. The experimental conditions should consequently be interpreted as structured visual–acoustic scene conditions rather than purified manipulations of two independent environmental variables. Subsequent inferential and response-surface results were interpreted as sample-specific parameter tendencies and exploratory design references, not as causal effects or universal thresholds.

### 2.5 Experimental design and procedure

The VR experiment was designed to examine whether adding the actual acoustic context to the same campus visual scenes changed short-term restorative responses. A repeated-measures crossover design was used so that each participant could contribute responses under both visual-only and audiovisual conditions, thereby reducing between-participant variability. A total of 40 university students aged 18–28 years were initially recruited (mean age = 22.1 ± 2.3 years), including 20 female and 20 male participants. Participants represented engineering (*n* = 18, 45.0%), natural-science (*n* = 10, 25.0%), and humanities (*n* = 12, 30.0%) backgrounds. Seven participants (17.5%) reported prior experience with immersive VR. All participants had normal or corrected-to-normal vision and reported no colour blindness, hearing impairment, cardiovascular, neurological, or psychiatric disorders, or medication use that could affect autonomic nervous system responses. Before the experiment, participants were instructed to avoid alcohol within 24 h, caffeine and nicotine within 4 h–6 h, and insufficient sleep. The experimental protocol was reviewed and approved by the review committee (SUST20260108). Written informed consent was obtained from all participants before participation, and all participants received compensation for their participation.

No formal *a priori* power analysis was conducted before recruitment. The target sample of 40 participants was determined with reference to the repeated-measures crossover design, sample sizes used in comparable immersive-VR studies, and an allowance for anticipated data loss associated with VR intolerance, eye-tracking calibration, and physiological-signal recording (Mostajeran et al., 2023; Serra et al., 2025).

Because the study used a two-period crossover design, participants were assigned to presentation sequences rather than independent experimental groups. Twenty participants were initially assigned to the visual-only → audiovisual sequence and 20 to the audiovisual → visual-only sequence. Five participants were excluded from the final analytical sample: two because of VR-induced cybersickness, two because of unusable electrodermal recordings, and one because reliable eye-tracking data could not be obtained because of calibration/tracking failure. The final analytical sample therefore comprised 35 participants, with 18 remaining in the visual-only → audiovisual sequence and 17 in the audiovisual → visual-only sequence. The valid participant-level sample size was *n* = 35 for eye-tracking, EDA, PPG/HRV, and questionnaire outcomes. Demographic characteristics of the initially recruited and final analytical samples are compared in Supplementary Table S1. Participant flow, crossover-sequence allocation, and the final analytical sample are summarised in Figure 4.

**Figure 4:**
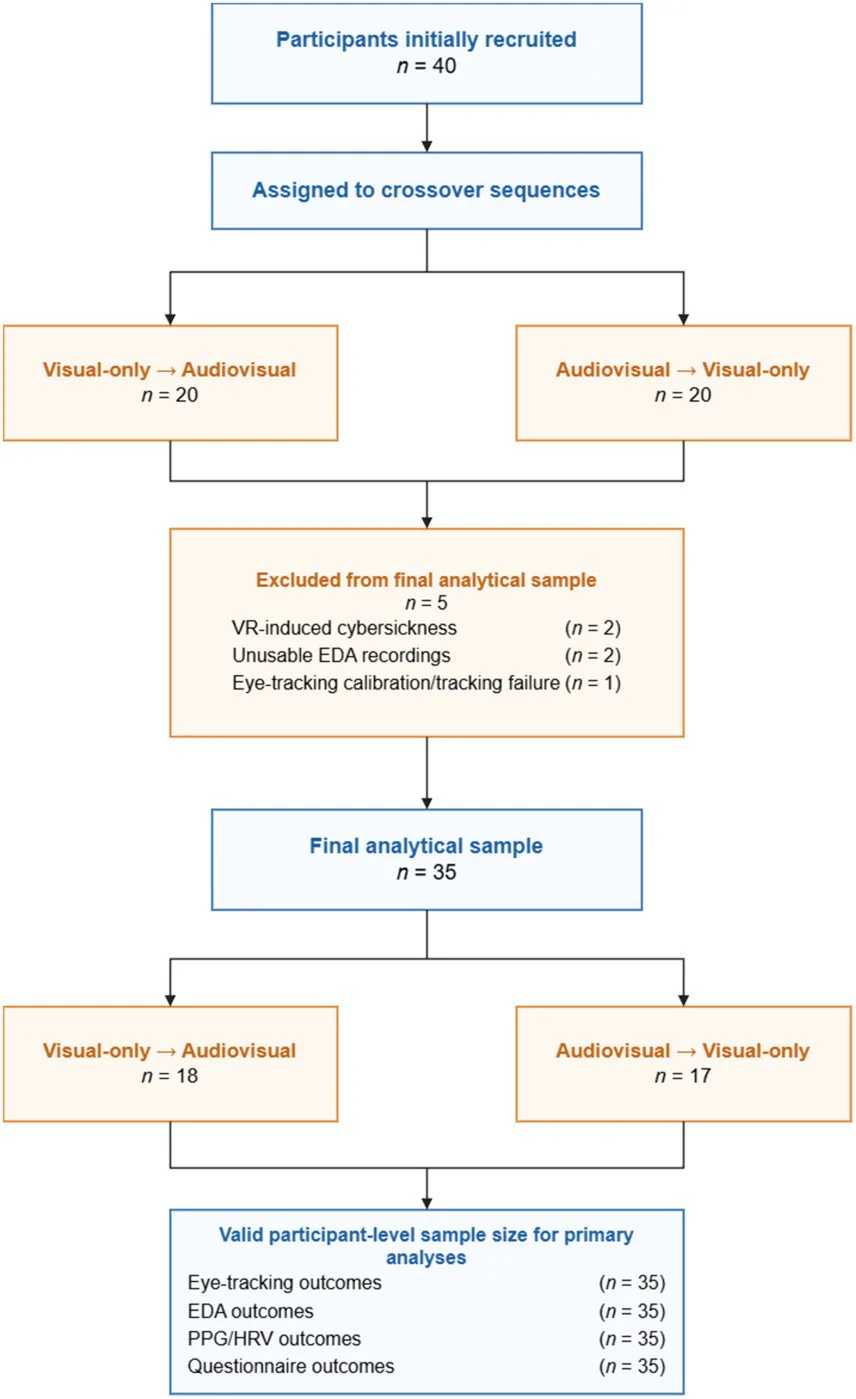
Participant flow, crossover-sequence allocation, exclusions, and final analytical sample.

The repeated-measures design yielded up to 630 participant-level exposure observations (35 participants × 9 scenes × 2 presentation conditions). These observations should not be interpreted as 630 independent environmental replicates; rather, they comprised repeated participant responses to nine distinct campus scene conditions presented under two sensory modes. Dependence among repeated observations from the same participant was accounted for in the mixed-effects analyses, while the limited scene-level replication was explicitly considered when interpreting NEFD- and GSI-related results.

An HTC VIVE Pro Eye headset was used to present the visual and audiovisual stimuli and to record gaze position, pupil diameter, fixation behaviour, and saccades. VR scenes were developed in Unity by mapping each panoramic image onto a virtual spherical environment. In the audiovisual condition, the corresponding 3-min field-recorded audio segment was presented synchronously through the VR headset headphones. In the visual-only condition, participants viewed the same panoramic scenes without field audio. The experimental setup and equipment configuration are shown in Table 3.

**Table 3:** Experimental setup and equipment configuration.

Experimental setup	Application	Equipment	Basic parameters	
			Frequency	Resolution
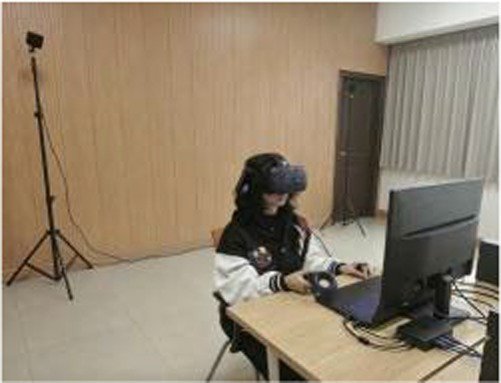	VR scene presentation	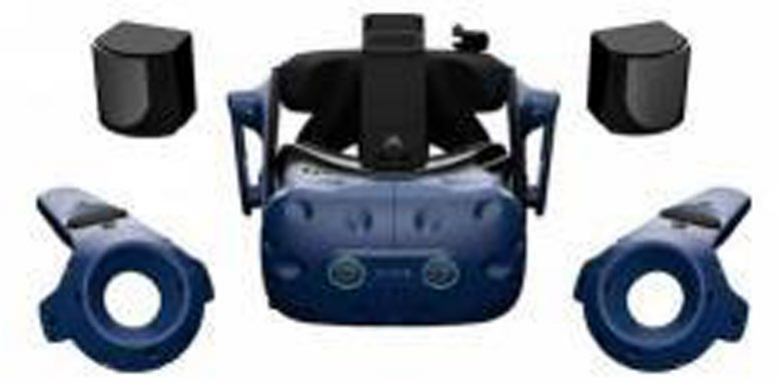	90 Hz display refresh rate	1,440 × 1,600 pixels per eye; 2,880 × 1,600 pixels combined
	Eye-tracking recording		120 Hz gaze-data output frequency	–
	Electrodermal and PPG recording	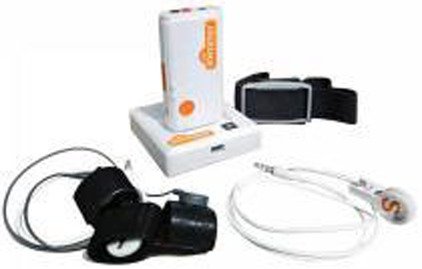	*Frequency*	*Bandwidth*
			128 Hz	DC–15.9 Hz

Physiological data were collected using a Shimmer3 GSR + module. Electrodermal activity was recorded through the GSR/EDA channel, and photoplethysmography signals were recorded through the PPG channel. Unity event markers were used to synchronise stimulus presentation, eye-tracking data, and physiological recordings.

A two-period crossover design compared the two presentation conditions, visual-only and audiovisual, while reducing individual and order effects. Participants were randomly assigned to one of two presentation sequences: visual-only followed by audiovisual, or audiovisual followed by visual-only (Lim and In, 2021). A 7-day washout period was included between sessions to reduce potential carryover effects. Within each session, the nine scenes were presented in a counterbalanced order using a Latin-square arrangement (Reese, 1997). The nine trials were divided into a first block of four scenes and a second block of five scenes, with a 20-min seated rest period between blocks to reduce fatigue and cumulative physiological carryover.

Presentation condition referred to the sensory mode through which the same VR scene was experienced. The visual-only condition presented the panoramic campus scene without the corresponding field recording, whereas the audiovisual condition paired the same visual scene with its matched 3-min sound clip. This contrast made it possible to test whether adding the actual acoustic context changed restorative responses beyond the visual scene alone. The crossover arrangement also reduced between-participant variability because each participant contributed responses under both sensory modes. Sequence assignment counterbalanced potential order effects, while the substantive comparison remained the difference between visual-only and audiovisual exposure. Each trial included a 3-min baseline, a 3-min mental-arithmetic stress-induction task, a 3-min VR exposure, approximately 2 min of subjective assessment, and approximately 2 min of inter-trial rest. Eye-tracking, EDA, and PPG signals were recorded during exposure. The overall experimental protocol is shown in Figure 5.

**Figure 5:**
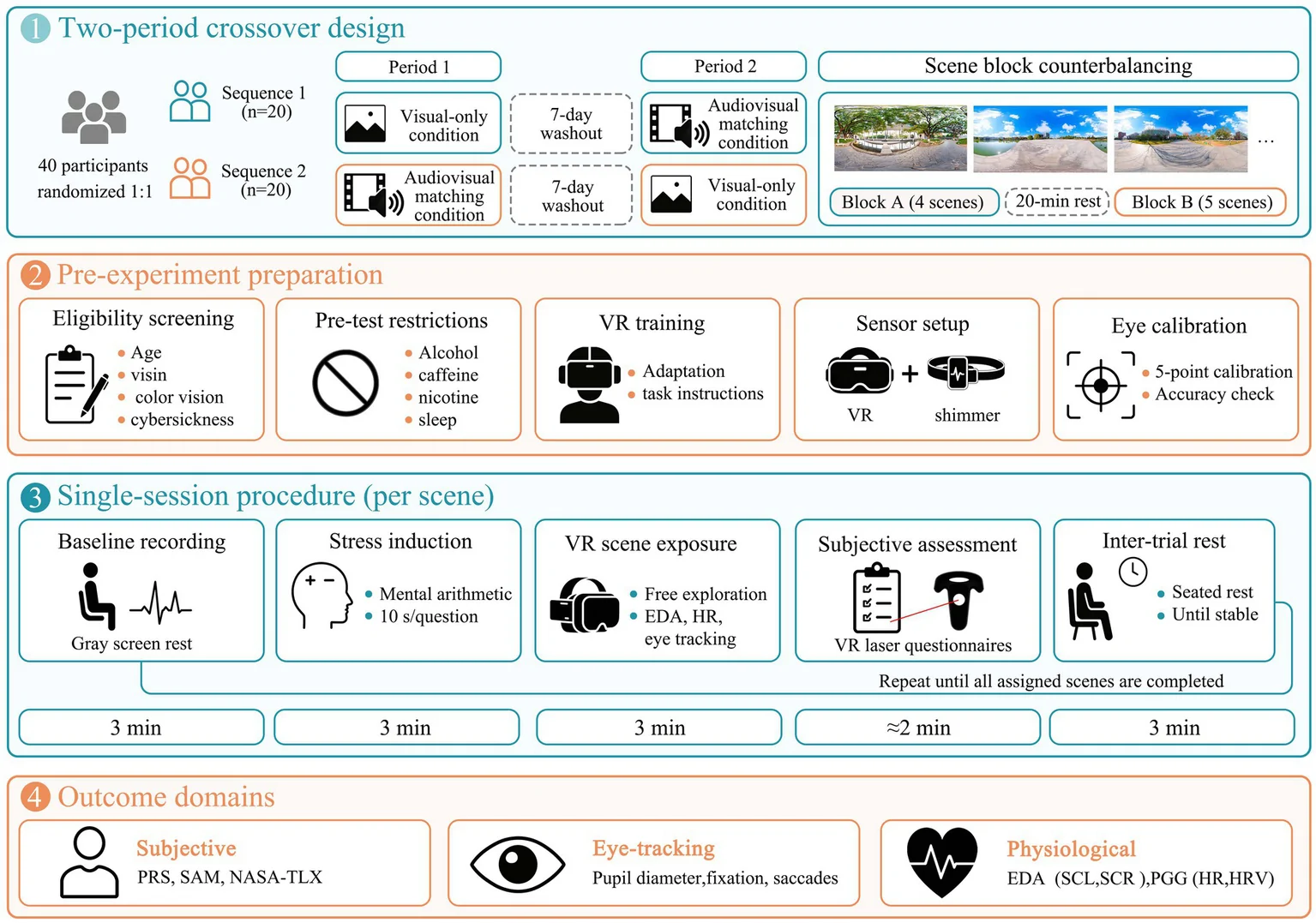
Experimental protocol and outcome measures.

### 2.6 Outcome measures and data preprocessing

The outcome measures were organised to capture four related dimensions of short-term environmental response: subjective experience, visual-cognitive processing, sympathetic arousal, and cardiovascular regulation. Subjective indicators included perceived restoration, emotional state, and self-reported cognitive load. These were assessed with the Perceived Restorativeness Scale (PRS), the Self-Assessment Manikin (SAM), and selected NASA-TLX dimensions, respectively (Hartig et al., 1997; Bradley and Lang, 1994; Hart and Staveland, 1988).

Eye-tracking indicators included mean pupil diameter, fixation count, saccade count, and saccade frequency (Duchowski, 2017). Mean pupil diameter was treated as a pupil-related indicator of processing demand and mental effort, while fixation and saccade measures described visual-search effort and scanning behaviour during VR exposure. Because pupil diameter can respond to both cognitive effort and visual luminance, the panoramic stimuli were adjusted to reduce between-scene differences in mean luminance and contrast while preserving their natural colour appearance and spatial structure (van der Wel and van Steenbergen, 2018; Jayawardena et al., 2025). Before the experiment, the nine VR panoramic images were luminance-matched. Each panorama was converted from sRGB to linear RGB, and spherical-area-weighted mean relative luminance was calculated to account for the equirectangular projection. The target luminance was set to 0.395, corresponding to the mean spherical-area-weighted linear relative luminance of the original stimulus set. Each panorama was exposure-scaled in linear RGB space to approach this target. After adjustment, the spherical-area-weighted mean relative luminance values of the nine final stimuli ranged from 0.3930 to 0.3953 (mean ± SD = 0.3946 ± 0.0008), indicating minimal residual between-scene luminance variation. Because absolute headset luminance was not measured photometrically, these values represent image-based relative luminance rather than physical display luminance in cd/m^2^. The individual mean relative luminance values for the nine final VR stimuli are provided in Supplementary Table S2. Pupil responses were analysed as within-trial changes relative to the preceding stress-induction stage rather than as raw absolute pupil size. Pupil-related changes were interpreted together with fixation, saccade, electrodermal, and subjective indicators rather than as standalone evidence of restoration.

Physiological indicators included skin conductance level (SCL), skin conductance response count (SCR count), skin conductance response amplitude (SCR amplitude), heart rate (HR), SDNN, RMSSD, and LF/HF. EDA preprocessing, PPG peak detection, and inter-beat-interval construction were performed using NeuroKit2 (Makowski et al., 2021), while HRV indicators were calculated using the pyHRV package (Gomes et al., 2019). EDA indicators were interpreted as measures of sympathetic arousal (Boucsein, 2012), whereas cardiovascular indicators provided complementary information on autonomic regulation.

Data-quality screening was conducted before the final statistical analysis. A participant-level exclusion strategy was applied to maintain a consistent analytical sample across the primary outcome domains. Participants were excluded if they were unable to complete the protocol because of VR-induced cybersickness or if a key recording modality contained substantial unusable data. Following these exclusions, the final sample of 35 participants provided valid eye-tracking, EDA, PPG/HRV, and questionnaire data for the primary analyses. Short transient signal losses due to blinking, headset movement, or sensor contact were handled during modality-specific preprocessing and did not result in additional participant-level exclusions.

For eye-tracking and physiological indicators, recovery responses were calculated as the difference between the VR exposure stage and the preceding stress-induction stage, as shown in Equation 3.

$$\begin{array}{c} \mathrm{\Delta Y}=Y_{\text{exposure}}-Y_{\text{stress}} \end{array}$$ (3)

where *Y*_exposure_ and *Y*_stress_ denote the mean values of the same indicator during VR exposure and stress induction, respectively. For pupil-related processing-demand and electrodermal-arousal indicators, lower or more negative change values generally indicated reduced activation relative to the stress-induction stage. Cardiovascular indicators were interpreted according to the physiological meaning of each individual measure rather than according to a single directional rule. Subjective indicators were analysed as post-exposure evaluations.

### 2.7 Statistical analysis

The statistical analysis was organised around the three primary hypotheses proposed in the Introduction, with linear mixed-effects models used as the primary participant-level analytical approach and response-surface modelling used as an exploratory tool for design-oriented interpretation. No single primary endpoint was formally preregistered or designated before data collection. Multiplicity was controlled using the Benjamini-Hochberg false discovery rate (FDR) procedure at the level of predefined outcome domains. Specifically, FDR correction was applied separately within each theoretically defined response family: subjective ratings, eye-tracking indicators, electrodermal activity indicators, and cardiovascular/HRV indicators. Within each domain, the *p* values from the omnibus fixed-effect tests in the linear mixed-effects models were adjusted together, including the main effects of presentation condition, NEFD, and GSI and their interaction terms. FDR-adjusted *q* < 0.05 was considered statistically supported. Follow-up simple effects and pairwise contrasts were conducted only to interpret omnibus effects that remained statistically supported after domain-wise FDR correction, and these pairwise comparisons were adjusted using the Tukey method.

A domain-wise rather than global FDR correction was used because the study treated subjective, eye-tracking, electrodermal, and cardiovascular outcomes as distinct but complementary response systems rather than as interchangeable indicators of a single endpoint. These domains differ in physiological meaning, expected temporal sensitivity, and measurement scale. Accordingly, multiplicity control was defined within substantively coherent outcome families so that statistical inference remained aligned with the study’s multisystem conceptual framework. This approach controlled the false discovery rate within each predefined domain while avoiding an unnecessarily conservative correction across heterogeneous response systems.

To address the absence of a formal *a priori* power analysis, a post-hoc sensitivity analysis was conducted for two representative psychophysiological outcomes, mean pupil diameter and skin conductance level (SCL). This analysis did not estimate observed post-hoc power from the obtained *p* values. Instead, it estimated the minimum detectable effect (MDE) under the final experimental design. For pupil diameter and SCL, simulation-based analyses preserved the final repeated-measures structure, including 35 participants, 629 complete recovery-minus-stress exposure observations, a participant-level random intercept, and variance components estimated from the corresponding fitted mixed-effects models. The same fixed-effect structure used in the primary analysis was retained: presentation condition × NEFD level × GSI level. Synthetic datasets were generated while systematically varying the magnitude of the target fixed-effect difference. For each candidate effect size, 5,000 simulated datasets were generated, and power was estimated as the proportion of simulations in which the prespecified target fixed-effect contrast reached statistical significance at *α* = 0.05. Approximate omnibus sensitivity for fixed effects with one, two, and four numerator degrees of freedom was additionally estimated using the noncentral F distribution and expressed as minimum detectable partial *η*^2^ and Cohen’s *f*. The analysis distinguished between participant-level repeated observations and scene-level environmental exposure units. Participants contributed repeated responses across presentation conditions and scenes, whereas the nine real campus scenes constituted the distinct environmental exposure conditions represented in the experiment. Accordingly, the repeated exposure trials should not be interpreted as independent environmental replicates.

Linear mixed-effects models were fitted to examine response differences associated with presentation condition, NEFD level, GSI level, and their interactions while accounting for repeated measurements within participants (Bates et al., 2015). Presentation condition was treated as a categorical factor, and NEFD and GSI were treated as categorical within-subject factors with three levels (low, medium, and high). Participant identity was included as a random intercept. The general linear mixed-effects model structure is shown in Equation 4.

$$\begin{array}{c} \begin{array}{l} \text{Response}\sim \text{Presentationcondition}\times \\ \text{NEFD}\times \mathrm{GSI}+(1|\text{Participant}) \end{array} \end{array}$$ (4)

Because each NEFD–GSI combination was represented by only one real scene, scene identity was fully aligned with the corresponding NEFD–GSI cell and could not be estimated as an independently replicated scene-level factor. The mixed-effects models therefore account for dependence among repeated observations within participants, but they do not provide independent replication at the scene level. Repeated observations from the same 35 participants improve the estimation of within-participant response differences but cannot substitute for independent environmental replication. Consequently, the models cannot statistically separate NEFD- or GSI-related response differences from unmeasured scene-level characteristics. NEFD, GSI, and their interaction are therefore interpreted only as exploratory contrasts among the nine structured audiovisual scene conditions, rather than as generalisable or independently replicated effects attributable specifically to the two environmental indicators.

Subjective, eye-tracking, electrodermal, and cardiovascular indicators were analysed using the same participant-level model structure. Interaction terms that remained statistically supported after domain-wise FDR correction were examined using estimated marginal means, and pairwise contrasts were adjusted for multiple comparisons using the Tukey method (Lenth, 2026). These analyses were used to characterise response differences across the sampled presentation and NEFD–GSI scene conditions, with interpretation restricted to the nine scenes represented in the experiment.

Potential order and carryover effects were mitigated through the experimental design rather than modelled directly. Participants were randomly assigned to two presentation sequences, the two sessions were separated by a 7-day washout interval, and scene order within each session was counterbalanced using a Latin-square arrangement. The primary mixed-effects models therefore focused on presentation condition and the structured NEFD–GSI scene conditions, with participant identity included as a random intercept to account for repeated measurements.

Response-surface modelling was used to visualise NEFD–GSI parameter combinations associated with more favourable response patterns (Myers et al., 2016). Estimated marginal means from the mixed-effects models were used as inputs for second-order polynomial response-surface models. The resulting surfaces illustrated nonlinear tendencies and potentially favourable audiovisual parameter zones. Because the response surfaces were derived from nine representative NEFD–GSI scene conditions, they were interpreted as exploratory descriptions within the sampled parameter range rather than as validated optimisation models or universal design thresholds.

The two analytical components therefore served different but connected purposes. Mixed-effects models provided the primary inferential evidence for response differences among the sampled visual–acoustic conditions. Response-surface models supported design-oriented interpretation by visualising response tendencies across the observed NEFD–GSI parameter space.

## 3 Results

The results are presented in four stages that reflect the analytical hierarchy of the study. First, the reliability of the automated environmental indicators is evaluated to determine whether NEFD and GSI are suitable for relative comparison among the sampled scenes. Second, mixed-effects models provide the primary inferential evidence on the associations of presentation condition, NEFD, GSI, and their interactions with psychophysiological response indicators, with Benjamini–Hochberg FDR correction applied separately within predefined outcome domains. Third, subjective evaluations are examined alongside eye-tracking and physiological response patterns to assess cross-system convergence. Finally, exploratory response-surface modelling visualises NEFD-GSI response tendencies and supports cautious design translation within the sampled parameter range. Post-hoc sensitivity estimates are then reported to contextualise the selective significant findings and the limited differentiation observed in cardiovascular outcomes.

### 3.1 Reliability of automated audiovisual indicators

Manual validation supported the use of NEFD and GSI as relative environmental measures for comparing the sampled audiovisual scenes. For the visual indicator, the manually checked natural-element masks showed acceptable-to-good agreement with the automated segmentation results, with IoU = 0.75 and Dice = 0.87. NEFD values derived from the automated masks were strongly correlated with those recalculated from the manually checked masks (*ρ* = 0.84, *p* < 0.01). The low–medium–high NEFD classification also showed substantial agreement with the manually checked reference classification (weighted *κ* = 0.72). Most discrepancies involved thin branches, distant vegetation, and boundary pixels between natural and built elements. Although the automated segmentation did not reproduce every natural-element boundary perfectly, the overall NEFD pattern remained sufficiently stable for relative comparison among the sampled scenes.

For the acoustic indicator, agreement between human-labelled sound categories and model-derived dominant categories was moderate to substantial (Cohen’s *κ* = 0.66). Segment-level manual soundscape-naturalness ratings were positively associated with segment-level GSI values (*ρ* = 0.69, *p* < 0.01). These results indicate that the model-derived GSI captured meaningful variation in the relative dominance of natural sound components across the experimental stimuli, although it should not be interpreted as a complete representation of perceived soundscape quality.

Taken together, the validation results supported the use of NEFD and GSI as sample-relative environmental indicators in the subsequent mixed-effects and exploratory response-surface analyses.

### 3.2 Associations of presentation condition, NEFD, and GSI with psychophysiological responses

Linear mixed-effects models examined the associations of presentation condition, NEFD, GSI, and their interactions with short-term psychophysiological responses. Core fixed-effect statistics are summarised in Table 4, with Benjamini–Hochberg FDR correction applied within predefined outcome domains. Given the absence of independent scene replication within NEFD–GSI cells, these tests are interpreted as contrasts among the nine sampled scene conditions rather than as evidence of generalisable scene-level effects attributable independently to NEFD or GSI.

**Table 4:** Summary of core NEFD, GSI, and NEFD × GSI fixed effects from linear mixed-effects models of psychophysiological responses.

Domain	Outcome	NEFD	GSI	NEFD × GSI
Cognitive	Pupil diameter	*F*(2, 578) = 15.03; *q* < 0.001; *ηp*^2^ = 0.049	*F*(2, 578) = 241.15; *q* < 0.001; *ηp*^2^ = 0.455	*F*(4, 578) = 7.41; *q* < 0.001; *ηp*^2^ = 0.049
Cognitive	Saccade frequency	*F*(2, 578) = 6.95; *q* = 0.003; *ηp*^2^ = 0.024	*F*(2, 578) = 81.31; *q* < 0.001; *ηp*^2^ = 0.220	*F*(4, 578) = 3.47; *q* = 0.019; *ηp*^2^ = 0.024
Cognitive	Saccade count	*F*(2, 578) = 12.89; *q* < 0.001; *ηp*^2^ = 0.043	*F*(2, 578) = 145.99; *q* < 0.001; *ηp*^2^ = 0.336	*F*(4, 578) = 3.89; *q* = 0.010; *ηp*^2^ = 0.026
Cognitive	Fixation count	*F*(2, 578) = 13.12; *q* < 0.001; *ηp*^2^ = 0.043	*F*(2, 578) = 148.00; *q* < 0.001; *ηp*^2^ = 0.339	*F*(4, 578) = 3.94; *q* = 0.010; *ηp*^2^ = 0.027
EDA	SCL	*F*(2, 578) = 2.23; *q* = 0.286; *ηp*^2^ = 0.008	*F*(2, 578) = 14.85; *q* < 0.001; *ηp*^2^ = 0.049	*F*(4, 578) = 2.65; *q* = 0.157; *ηp*^2^ = 0.018
EDA	SCR count	*F*(2, 578) = 1.08; *q* = 0.649; *ηp*^2^ = 0.004	*F*(2, 578) = 2.97; *q* = 0.157; *ηp*^2^ = 0.010	*F*(4, 578) = 5.56; *q* = 0.001; *ηp*^2^ = 0.037
EDA	SCR amplitude	*F*(2, 578) = 0.08; *q* = 0.938; *ηp*^2^ = 0.000	*F*(2, 578) = 1.26; *q* = 0.598; *ηp*^2^ = 0.004	*F*(4, 578) = 2.45; *q* = 0.157; *ηp*^2^ = 0.017
PPG/HRV	HR	*F*(2, 578) = 1.14; *q* = 0.952; *ηp*^2^ = 0.004	*F*(2, 578) = 1.45; *q* = 0.943; *ηp*^2^ = 0.005	*F*(4, 578) = 0.90; *q* = 0.952; *ηp*^2^ = 0.006
PPG/HRV	SDNN	*F*(2, 578) = 0.29; *q* = 0.952; *ηp*^2^ = 0.001	*F*(2, 578) = 0.78; *q* = 0.952; *ηp*^2^ = 0.003	*F*(4, 578) = 0.76; *q* = 0.952; *ηp*^2^ = 0.005
PPG/HRV	RMSSD	*F*(2, 578) = 0.19; *q* = 0.952; *ηp*^2^ = 0.001	*F*(2, 578) = 0.27; *q* = 0.952; *ηp*^2^ = 0.001	*F*(4, 578) = 0.50; *q* = 0.952; *ηp*^2^ = 0.004
PPG/HRV	LF/HF	*F*(2, 578) = 0.03; *q* = 0.966; *ηp*^2^ = 0.000	*F*(2, 578) = 0.20; *q* = 0.952; *ηp*^2^ = 0.001	*F*(4, 578) = 12.01; *q* < 0.001; *ηp*^2^ = 0.077

For the eye-tracking outcomes, NEFD, GSI, and the NEFD × GSI interaction remained statistically significant after FDR correction for mean pupil diameter, saccade frequency, saccade count, and fixation count. For mean pupil diameter, significant effects were observed for NEFD, *F*(2, 578) = 15.028, *q* < 0.001, partial *η*^2^ = 0.0494; GSI, *F*(2, 578) = 241.150, *q* < 0.001, partial *η*^2^ = 0.4549; and the NEFD × GSI interaction, *F*(4, 578) = 7.411, *q* < 0.001, partial *η*^2^ = 0.0488. Similar FDR-supported NEFD, GSI, and NEFD × GSI effects were observed for saccade frequency, saccade count, and fixation count. In contrast, presentation condition alone did not show a significant main effect on these eye-tracking outcomes.

Electrodermal responses showed a more selective pattern. For SCL, the GSI main effect remained significant after FDR correction, *F*(2, 578) = 14.854, *q* < 0.001, partial *η*^2^ = 0.0489. Although the NEFD × GSI term reached nominal significance for SCL (*p* = 0.032), it did not remain significant after FDR correction (*q* < 0.157). For SCR count, the NEFD × GSI interaction remained significant, *F*(4, 578) = 5.563, *q* = 0.001, partial *η*^2^ = 0.0371, whereas the corresponding NEFD and GSI main effects were not statistically significant after correction. No core fixed effect for SCR amplitude remained significant after FDR correction. Additional condition-related interaction effects are reported in Supplementary Table S3.

Figure 6 summarises the overall distributions of standardised recovery changes across the three physiological systems. Considerable within-indicator variability was evident for the eye-tracking and electrodermal measures, whereas most cardiovascular indices were concentrated relatively close to zero. These descriptive distributions are broadly consistent with the inferential results, which indicated stronger differentiation in eye-tracking outcomes, more selective effects in electrodermal measures, and comparatively limited effects in cardiovascular indices.

**Figure 6:**
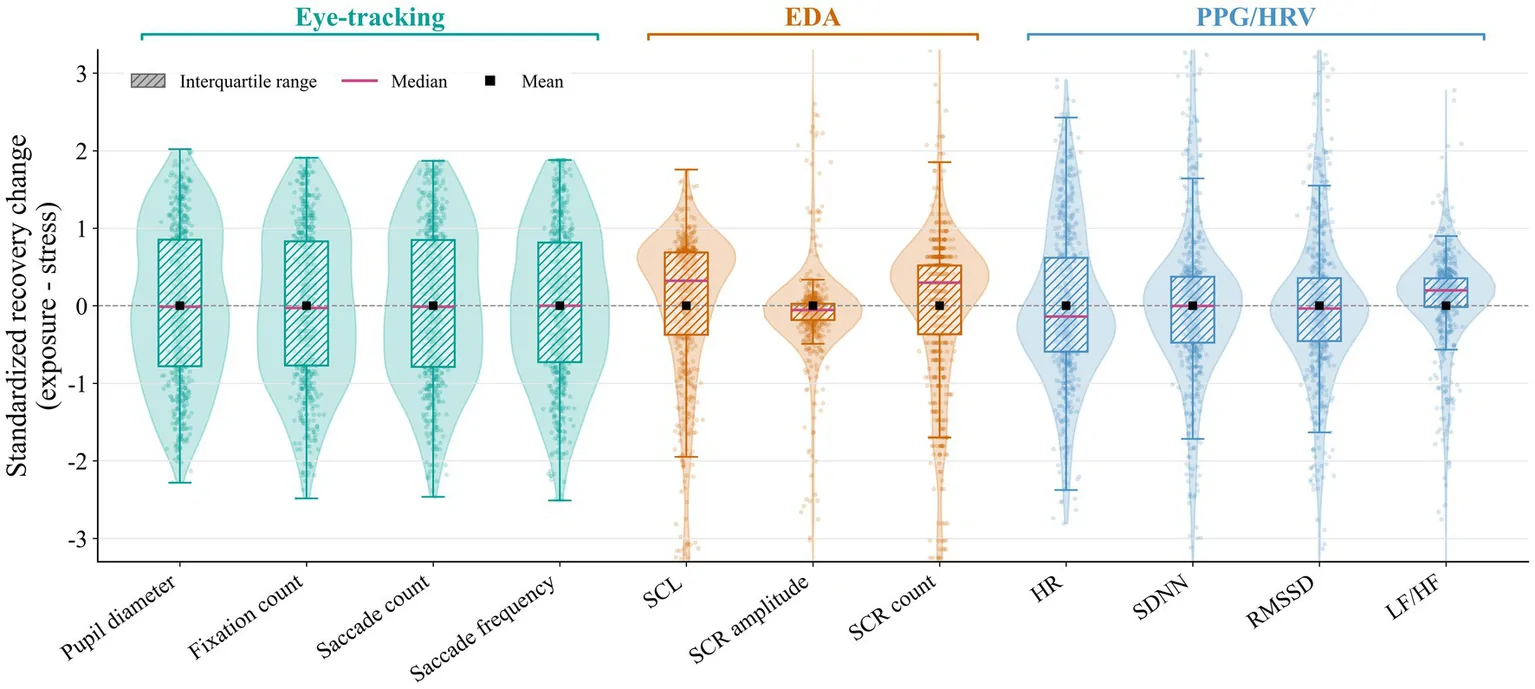
System-specific distributions of standardised recovery changes across eye-tracking, electrodermal, and cardiovascular indicators.

Cardiovascular outcomes showed comparatively limited sensitivity to the experimental conditions. HR, SDNN, and RMSSD showed no FDR-supported main effects of presentation condition, NEFD, or GSI and no significant NEFD × GSI interactions. LF/HF likewise showed no significant main effects, although its NEFD × GSI interaction remained significant, *F*(4, 578) = 12.011, *q* < 0.001, partial *η*^2^ = 0.0767. Accordingly, descriptive differences in HR and most HRV indices were small or inconsistent and were not interpreted as evidence of reliable cardiovascular restoration during the short exposure period.

Follow-up estimated marginal means and Tukey-adjusted pairwise contrasts were used to characterise the FDR-supported interaction effects. For pupil diameter, NEFD-related differences varied across GSI levels. Under medium GSI, the medium-NEFD condition showed a smaller pupil-diameter change than both the low-NEFD condition (low − medium = 0.452, 95% CI [0.352, 0.552], Tukey-adjusted *p* < 0.001) and the high-NEFD condition (medium − high = −0.166, 95% CI [−0.266, −0.067], *p* < 0.001). A similar pattern was observed under high GSI, where medium NEFD produced a smaller pupil-diameter change than low NEFD (low − medium = 0.490, 95% CI [0.390, 0.589], *p* < 0.001) and high NEFD (medium − high = −0.237, 95% CI [−0.337, −0.137], *p* < 0.001).

Similar context-dependent patterns were observed for saccade frequency, saccade count, and fixation count. Under high-GSI conditions, medium NEFD was associated with lower saccade frequency than both low NEFD (low − medium = 7.082, 95% CI [4.576, 9.587], *p* < 0.001) and high NEFD (medium − high = −3.836, 95% CI [−6.342, −1.330], *p* = 0.001). Comparable intermediate-NEFD patterns were also observed for saccade count and fixation count. For SCR count, NEFD differences were more selective: under high GSI, the medium-NEFD condition showed a greater reduction than the low-NEFD condition (low − medium = 7.043, 95% CI [4.112, 9.973], *p* < 0.001), whereas the corresponding contrasts under lower-GSI conditions were weaker or non-significant. Complete estimated marginal means, pairwise contrasts, 95% confidence intervals, and Tukey-adjusted *p* values are provided in Supplementary Table S4. Figure 7 illustrates these interaction patterns across the nine NEFD–GSI scene conditions.

**Figure 7:**
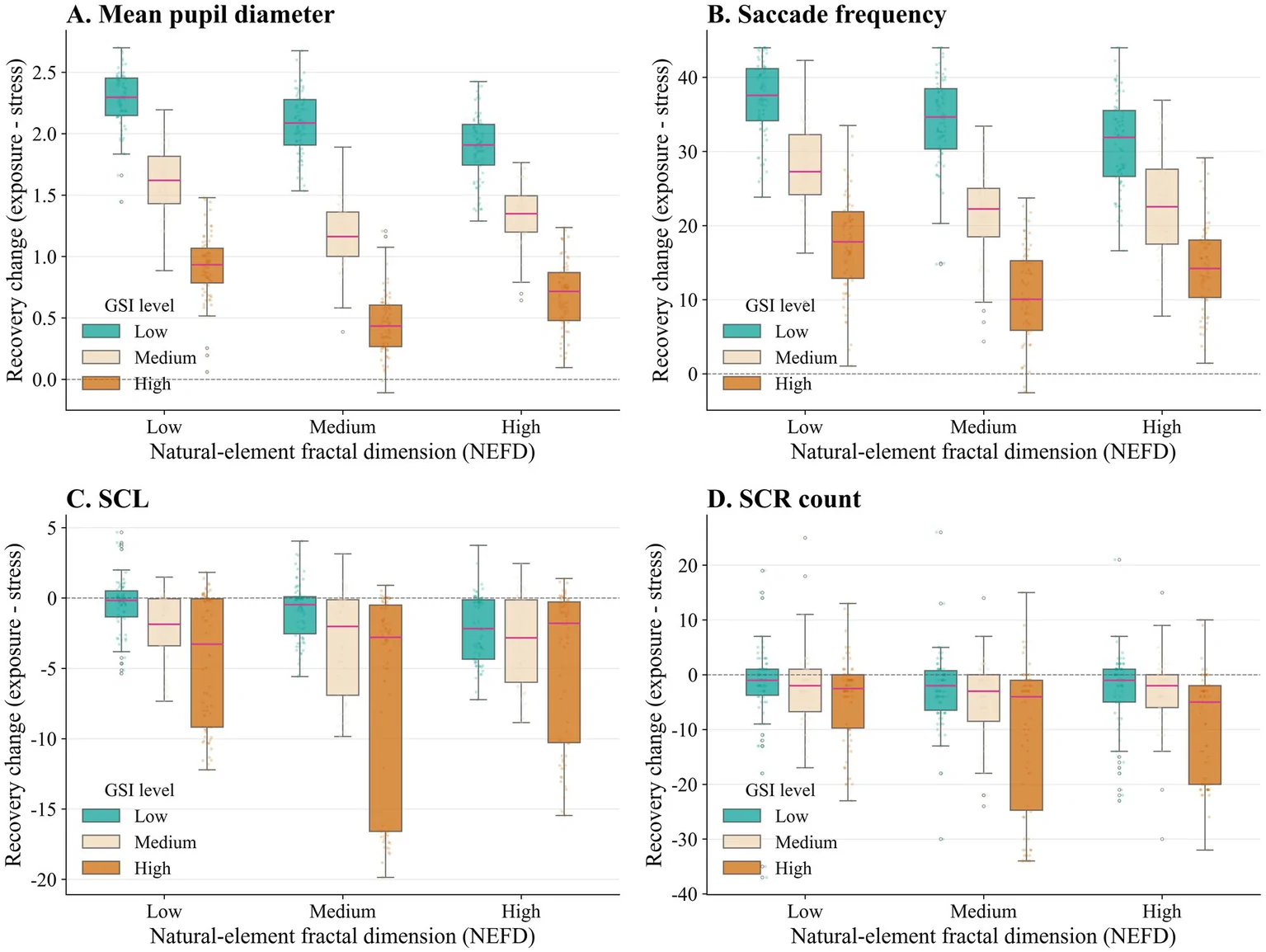
Distribution of recovery changes in selected eye-tracking and electrodermal indicators across NEFD–GSI combinations: **(A)** mean pupil diameter; **(B)** saccade frequency; **(C)** skin conductance level (SCL); and **(D)** skin conductance response (SCR) count.

To contextualise the selective significant findings and the largely non-significant cardiovascular outcomes, post-hoc sensitivity estimates were examined for two representative psychophysiological indicators, pupil diameter and SCL. For pupil diameter, the estimated participant random-intercept variance was 0.001909 and the residual variance was 0.066778. Under the observed repeated-measures structure, the minimum detectable effect at 80% power was approximately 0.122 mm (d-equivalent ≈ 0.47). For SCL, the corresponding participant random-intercept and residual variances were 3.342322 and 11.965159, respectively, yielding a minimum detectable effect of approximately 2.32 SCL units (d-equivalent ≈ 0.67). Approximate omnibus sensitivity analyses indicated minimum detectable partial *η*^2^ values of approximately 0.016 for effects with df1 = 2 and 0.020 for effects with df1 = 4.

Overall, the FDR-corrected omnibus tests and Tukey-adjusted simple-effect contrasts were consistent with H1 in showing broad associations between GSI and several eye-tracking and electrodermal outcomes. The recurrent intermediate-NEFD pattern under medium- and high-GSI conditions was consistent with the nonlinear interpretation underlying H2, while the clearer NEFD-related differentiation under higher-GSI conditions was consistent with the context-dependent pattern proposed in H3. These associations were strongest for eye-tracking outcomes and more selective for electrodermal measures, whereas cardiovascular responses showed substantially weaker and less consistent differentiation.

### 3.3 Convergence between subjective and psychophysiological responses

Subjective responses were analysed using linear mixed-effects models for PRS, SAM, and NASA-TLX indicators. Figure 8 summarises the fixed-effect test statistics for the main effects, two-way interactions, and three-way interaction, with statistical support evaluated using domain-wise FDR-adjusted q values. Subjective associations were heterogeneous across outcome dimensions: several PRS dimensions showed strong differentiation across the sampled visual–acoustic conditions, whereas SAM and NASA-TLX outcomes showed more selective associations. GSI was associated with multiple dimensions of perceived restoration, emotional response, and self-reported task demand, while selected NEFD-related and interaction terms were also supported for specific outcomes. Complete fixed-effect statistics for the subjective outcomes are provided in Supplementary Table S5.

**Figure 8:**
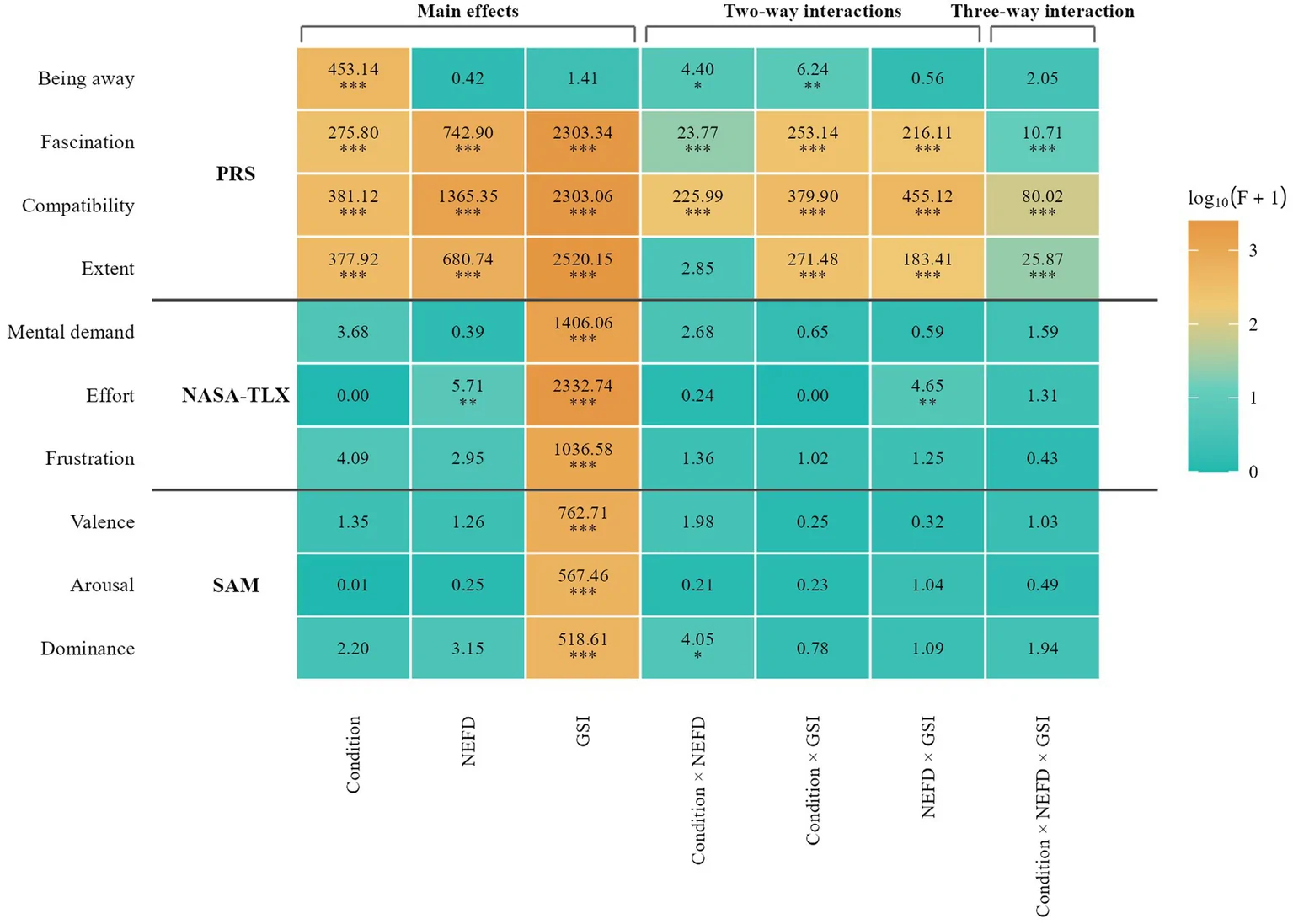
Fixed-effect results for subjective response indicators from linear mixed-effects models. Cell values indicate *F* statistics, and asterisks indicate significance based on domain-wise FDR-adjusted q values (*q < 0.05, **q < 0.01, ***q < 0.001). Colour intensity represents log10(F + 1).

For perceived restorativeness, fascination and compatibility showed broad FDR-supported associations with presentation condition, NEFD, GSI, and their interactions, whereas extent showed a similarly strong pattern except for the Condition × NEFD term. Being away was comparatively less sensitive to NEFD and GSI themselves, with significant effects occurring primarily for presentation condition and its interactions with NEFD and GSI. For emotional responses, GSI showed the most consistent associations, with significant GSI effects observed for SAM valence, arousal, and dominance; other terms were generally weak or non-significant apart from a Condition × NEFD interaction for dominance. Among the NASA-TLX dimensions displayed in Figure 8, GSI showed significant associations with mental demand, effort, and frustration, while additional NEFD and NEFD × GSI effects were observed for effort.

The subjective findings showed partial convergence with the eye-tracking and electrodermal results. GSI-related differentiation was particularly consistent across perceived restoration, emotional response, and workload measures, paralleling the broad GSI associations observed in several psychophysiological outcomes. By contrast, NEFD-related subjective responses were more selective and often appeared through specific main or interaction terms rather than as a uniform gradient. This convergence was therefore pattern-level rather than one-to-one across all response systems.

Subjective outcomes provided complementary rather than redundant information. PRS dimensions showed stronger and more complex differentiation than SAM and NASA-TLX measures, while eye-tracking and electrodermal indicators showed clearer short-term psychophysiological variation. Taken together, the results indicate partial cross-system consistency between perceived experience and short-term psychophysiological responses, with GSI showing the most consistent associations across domains.

### 3.4 Exploratory response-surface patterns

Response-surface modelling was used to visualise how selected psychophysiological response indicators varied across the sampled NEFD–GSI parameter space. The resulting surfaces were treated as exploratory interpolations derived from the nine representative scene conditions rather than as independently validated optimisation models. Eye-tracking indicators showed the clearest surface patterns, electrodermal indicators showed more selective tendencies, and cardiovascular indicators were less consistent. Accordingly, the surfaces were interpreted as sample-specific empirical response patterns within the observed parameter range.

As shown in Figure 9, GSI showed the clearest overall response gradient across the selected eye-tracking and electrodermal outcomes. Higher GSI was generally associated with smaller mean pupil-diameter changes, lower saccade frequency, greater SCL reduction, and lower SCR count. NEFD-related patterns were nonlinear and varied across GSI levels. Under lower-GSI conditions, differences across NEFD levels were generally less pronounced, whereas under medium- and high-GSI conditions, intermediate NEFD values were associated with more favourable response tendencies for several selected indicators. These patterns were not uniform across all outcomes and should therefore be interpreted as descriptive tendencies within the current nine-scene sample.

**Figure 9:**
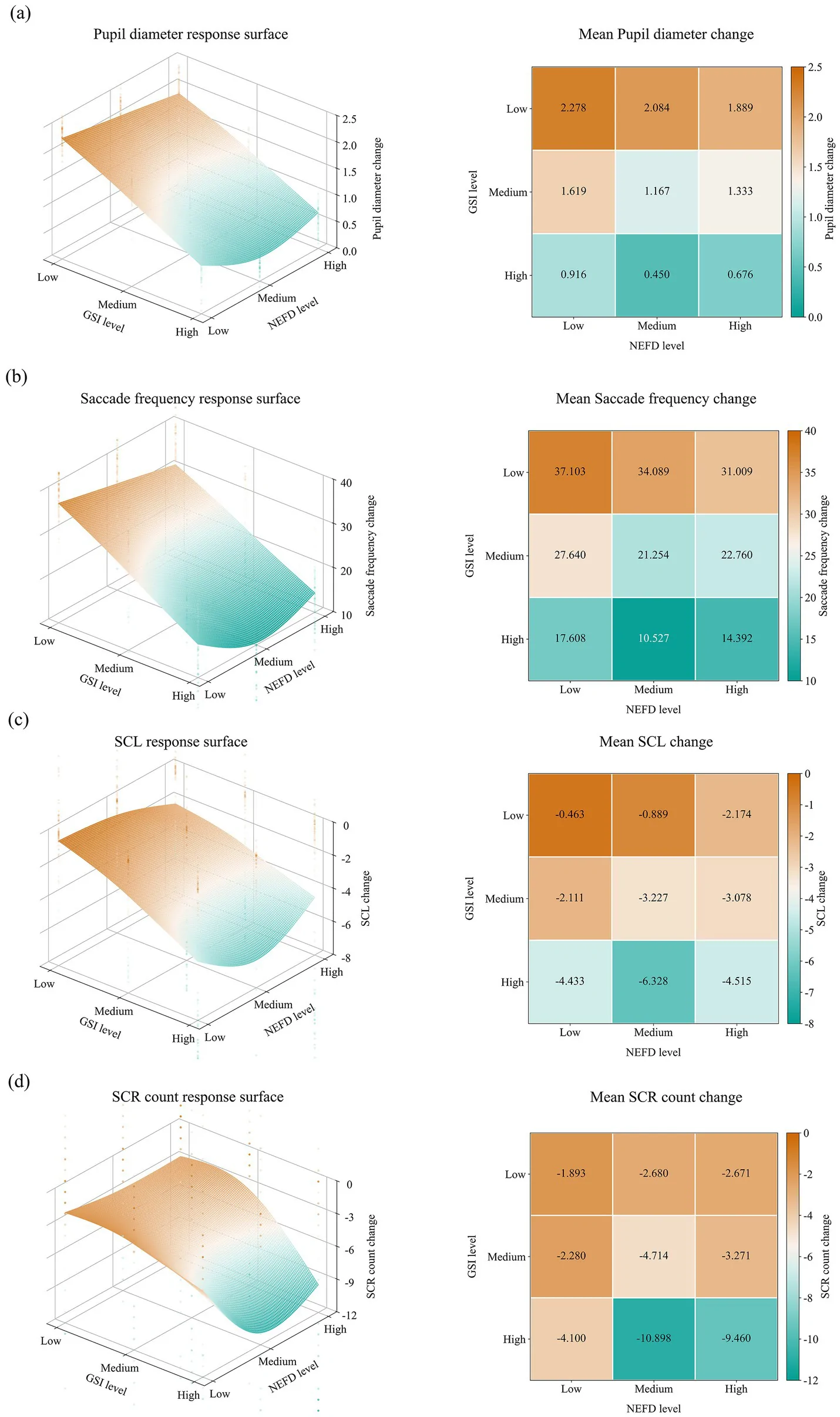
Exploratory response-surface patterns for selected psychophysiological response indicators. For each indicator, the left panel shows the fitted three-dimensional surface and the right panel shows the corresponding NEFD–GSI heatmap: **(a)** mean pupil diameter **(b)** saccade frequency **(c)** SCL, and **(d)** SCR count.

Across the selected eye-tracking and electrodermal indicators, one recurrent empirical pattern occurred in the region characterised by relatively high GSI and intermediate NEFD values, approximately GSI = 0.71–1.00 and NEFD = 1.31–1.52. A second, less consistent pattern was observed for selected outcomes under medium GSI and intermediate NEFD. These ranges describe response tendencies represented within the fitted surfaces and should not be interpreted as optimal parameter combinations or validated design thresholds. No single NEFD–GSI region showed uniformly favourable responses across all outcome domains.

The numerical ranges should therefore be interpreted only as empirical parameter regions observed within the sampled Suzhou campus scenes. Because the response surfaces were derived from nine representative visual–acoustic conditions without independent scene replication within each NEFD–GSI cell, they do not constitute validated optimisation models or transferable design thresholds. Further studies using multiple independently replicated scenes per parameter combination and external validation across campuses, climates, seasons, and user groups are required before these patterns can inform general design guidance.

## 4 Discussion

This study examined campus green spaces as multisensory components of health-supportive built environments rather than as visually green settings alone. The findings indicate four interrelated patterns. First, GSI showed the broadest and most consistent associations with short-term eye-tracking, electrodermal, and subjective responses. Second, natural-element complexity was related to restoration in a nonlinear and context-dependent manner, with medium-NEFD conditions showing favourable tendencies for several outcomes when supported by more natural soundscapes. Third, the response difference between visual-only and audiovisual exposure was not uniform, indicating that the contribution of matched acoustic information depended on the specific NEFD–GSI condition and response system. These findings support a multisensory and nonlinear interpretation of short-term restorative responses, while the limited scene sample and controlled VR protocol require cautious translation to real-world design.

### 4.1 The role of GSI in short-term restorative responses

GSI emerged as the most consistent acoustic indicator associated with short-term restorative responses in the sampled campus scenes. Across eye-tracking, electrodermal, and subjective indicators, higher GSI was generally associated with lower pupil-related processing demand, lower electrodermal activation, and more favourable subjective evaluations of restoration and affect. These findings reinforce the view that the restorative performance of campus green spaces cannot be understood through visible greenness or visual landscape structure alone. Acoustic source composition forms part of the environmental condition through which users experience courtyards, shaded walkways, building entrances, dormitory edges, and other frequently encountered campus spaces (Alvarsson et al., 2010; Ratcliffe, 2021; Uebel et al., 2021; Liu F. et al., 2022; Liu Q. et al., 2022).

The results extend greenness-oriented restorative-environment research by treating natural sound not simply as background context, but as a measurable component of multisensory environmental quality (Abbott et al., 2016; Krzywicka and Byrka, 2017; Kogan et al., 2018; Bai and Zhang, 2024; Fan and Baharum, 2024; Gilmour et al., 2024). Previous research has linked natural sounds and restorative soundscapes with attention restoration, stress reduction, mood recovery, perceived restorativeness, and broader well-being pathways (Bowler et al., 2010; White et al., 2013; Kuo, 2015; Twohig-Bennett and Jones, 2018). The present study adds a source-composition perspective by using GSI to represent the relative dominance of natural and anthropogenic sound components. Compared with NEFD, GSI showed broader and more stable associations across the measured response systems, suggesting that greater GSI-based natural sound-source dominance may provide a relatively consistent acoustic context within which visual landscape conditions are processed.

This interpretation is particularly relevant to university campuses, where green spaces are embedded among teaching buildings, residential areas, circulation routes, service spaces, mechanical equipment, and traffic interfaces. A visually green or structurally complex space may still provide limited restorative support when traffic, construction, mechanical ventilation, or dense social activity dominates the acoustic environment. Conversely, natural sound components may strengthen the experiential quality of visually supportive settings (Browning et al., 2019; Knaust et al., 2022; Jo and Jeon, 2024; Sella et al., 2025; Yoon and Jeon, 2025). The findings are consistent with evidence that landscape and soundscape characteristics jointly shape stress recovery, environmental preference, and perceived quality (Uebel et al., 2021; Liu F. et al., 2022; Liu Q. et al., 2022; Smalley et al., 2023). They further suggest that acoustic quality is a relevant consideration for campus landscape design and management, rather than merely an external disturbance assessed after visual landscape design has been completed.

### 4.2 Context-dependent role of natural-element complexity

Natural-element fractal complexity was associated with differences in short-term response, but the NEFD-related pattern was less stable than the GSI gradient and varied according to auditory context. Higher NEFD did not correspond uniformly to more favourable responses. Instead, medium-NEFD conditions showed favourable tendencies for several eye-tracking and electrodermal indicators when combined with medium or high GSI. The recurrent pattern under higher-GSI conditions suggests that natural visual complexity may be more relevant when the surrounding acoustic environment provides a supportive sensory context (Hagerhall et al., 2004; Stamps, 2004; Stevens, 2018; Liu et al., 2021; Robles et al., 2021).

NEFD should be interpreted as a structural descriptor rather than as a general measure of visual quality. It captures the contour-based fractal complexity of segmented vegetation, terrain, and water elements, but does not directly represent vegetation quantity, visible green proportion, openness, enclosure, maintenance quality, visual preference, or spatial legibility. The results therefore do not indicate that one level of NEFD is universally restorative. Rather, they suggest that the organisation of natural visual elements may contribute to response differentiation in ways that depend on other sensory and spatial conditions.

One possible interpretation is that restorative visual environments require a balance between perceptual richness and environmental clarity (Laumann et al., 2001; Han, 2003; Herzog et al., 2003; Stamps, 2004; Liu et al., 2021). Previous research has associated visual complexity, coherence, legibility, and preference with environmental evaluation, while also indicating that psychological responses are not necessarily maximised at the highest level of structural complexity (Hagerhall et al., 2004; Stevens, 2018; Robles et al., 2021). Within the sampled scenes, medium NEFD may have provided sufficient natural structure to support engagement without producing the same visual-processing demands associated with selected highly complex conditions. However, this interpretation remains provisional because coherence, legibility, and perceived disorder were not independently measured.

From a design perspective, these findings caution against equating restorative quality with maximum vegetation density or visual intricacy. Recent studies similarly suggest that greater vegetation exposure does not invariably produce stronger restorative benefits and that the spatial configuration of natural elements may be as important as their quantity (Lai et al., 2020; Liu et al., 2021; Shibata et al., 2024; Sun et al., 2024; Song et al., 2025). For campus landscapes, the more defensible implication is to consider layered vegetation, coherent blue–green composition, organised natural edges, and spatial legibility together rather than to maximise visual complexity as a single design objective. Any NEFD range observed in the present study should therefore be treated only as a sample-specific empirical reference and interpreted jointly with vegetation coverage, openness, enclosure, visibility, and soundscape conditions.

More broadly, the present pattern highlights the importance of considering potentially nonlinear relationships between environmental exposure and restorative response rather than assuming that “more nature” or “more complexity” necessarily produces greater benefit. In environmental-health and landscape-planning research, such nonlinear relationships are important because beneficial responses may increase across part of an exposure range, plateau beyond that range, or even reverse when sensory density, visual complexity, enclosure, or competing environmental demands become excessive. Identifying these transitions could help avoid both under-designed green infrastructure, in which natural exposure is insufficient to support restoration, and high-density or over-complexity conditions, in which additional vegetation or structural intricacy may increase perceptual demand without producing additional restorative benefit. From this perspective, the value of NEFD lies not in defining a universally desirable maximum, but in testing whether restorative responses vary across low, intermediate, and high levels under different soundscape conditions. However, the present study was not designed to establish a validated dose–response threshold. Given the limited number of representative scenes, the observed intermediate-NEFD tendency should instead be regarded as a hypothesis-generating empirical pattern requiring denser sampling and multiple independent scenes across comparable NEFD–GSI conditions.

### 4.3 Methodological implications of response-system sensitivity

The differing sensitivity of the response systems has methodological implications for short-duration VR studies. Eye-tracking indicators showed the clearest differentiation across NEFD–GSI conditions, followed by electrodermal indicators, whereas HR, SDNN, and RMSSD showed weaker or less consistent patterns. This does not imply that one response system is inherently more valid than another. Rather, different indicators operate over different temporal scales and capture different components of environmental response (Halbig and Latoschik, 2021; Amin and Faghih, 2022; Polo et al., 2022; Sánchez-Reolid et al., 2022; Long et al., 2023; Ferreira et al., 2024; Gorin et al., 2024; Yu et al., 2024; Liu and Zhou, 2025; Liu et al., 2025).

Cardiovascular indicators require more cautious interpretation under the 3-min exposure protocol. HR and HRV can be influenced by respiration, posture, baseline autonomic state, physical fitness, circadian variation, and individual recovery capacity (Halbig and Latoschik, 2021; Knaust et al., 2022; Yu et al., 2024). The weaker differentiation observed here should therefore not be interpreted as evidence that campus green spaces have no cardiovascular relevance. Instead, the exposure duration may have been more suitable for detecting rapid visual-cognitive and electrodermal changes than slower autonomic regulation. Future research could combine short-duration eye-tracking and EDA measures with longer exposures, controlled respiration, repeated visits, or longitudinal field monitoring to determine whether cardiovascular differences emerge over extended recovery periods. The post-hoc sensitivity analysis further suggests that weaker or null cardiovascular and electrodermal findings may partly reflect the detectable effect range of the present repeated-measures design, rather than the absence of any true environmental response.

### 4.4 Design-performance translation for campus green infrastructure

The present findings have potential implications for campus landscape assessment, but their translation should remain at the level of exploratory design reference rather than prescriptive optimisation. Across the nine sampled Suzhou campus scenes, more favourable short-term responses were repeatedly observed under conditions combining relatively high GSI with intermediate NEFD. The broader implication is not that campuses should reproduce a specific NEFD–GSI numerical combination, but that visual natural-element structure and soundscape composition should be considered jointly rather than optimised in isolation. The response-surface analysis identified a sample-specific empirical region corresponding approximately to GSI = 0.71–1.00 and NEFD = 1.31–1.52. These numerical ranges were interpolated from only nine representative Suzhou campus scenes and should not be interpreted as universal thresholds, optimal values, or transferable landscape-design standards. Figure 10 provides an exploratory conceptual mapping from the sample-specific high-GSI and intermediate-NEFD pattern to candidate campus landscape-design considerations. It is intended only as an interpretive illustration of possible design directions, rather than as a validated optimisation framework, a transferable threshold model, or a design standard.

**Figure 10:**
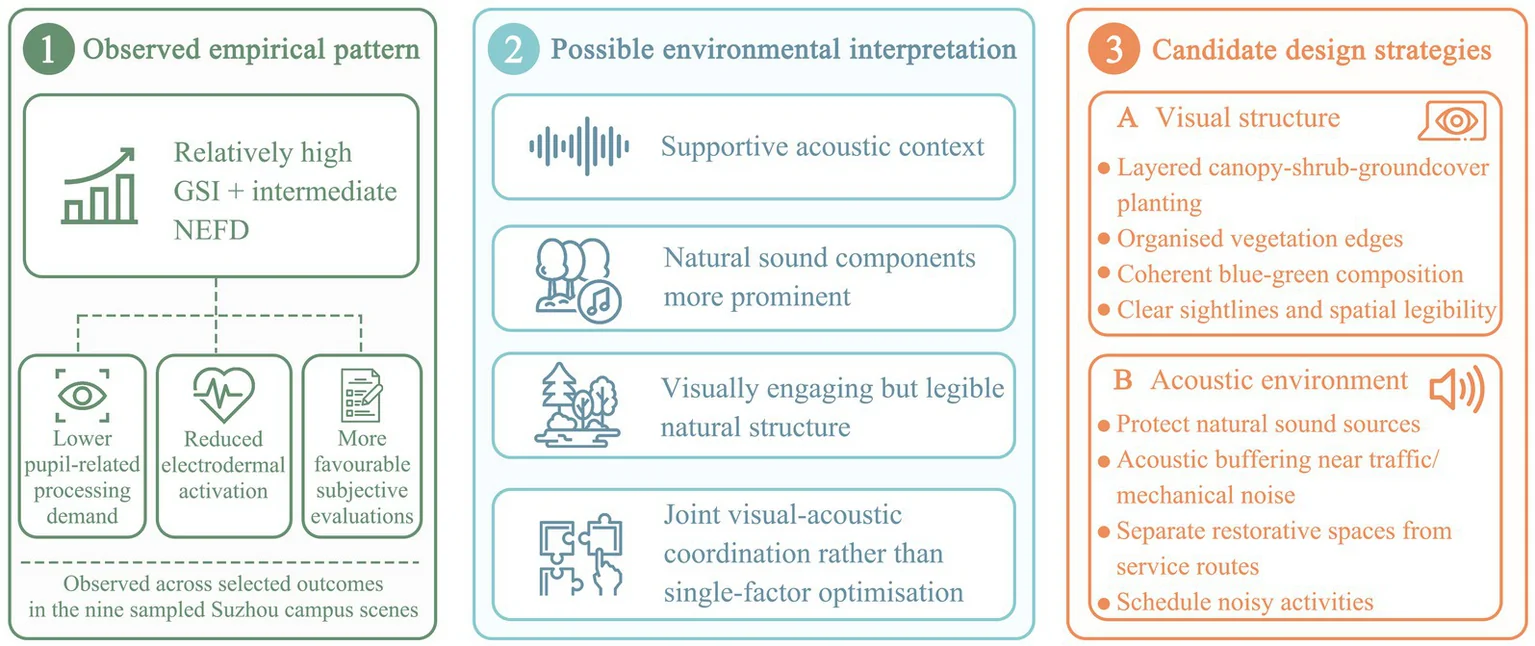
Exploratory conceptual mapping from observed NEFD–GSI response patterns to candidate campus design strategies.

From this perspective, acoustic quality should be considered in campus landscape design and management from the beginning rather than assessed only after visual design has been completed. Spaces intended for short breaks, informal study, waiting, circulation, and everyday recovery may benefit from preserving existing natural sound sources and supporting the ecological conditions that generate them (Alvarsson et al., 2010; Abbott et al., 2016; Krzywicka and Byrka, 2017; Ratcliffe, 2021; Uebel et al., 2021; Bai and Zhang, 2024; Fan and Baharum, 2024; Gilmour et al., 2024). Potential measures include habitat-supportive planting, protection of bird and insect activity, appropriate use of water features, separation of restorative spaces from traffic and service routes, acoustic buffering near mechanical equipment, and temporal management of noisy activities. These strategies should focus on improving the overall source composition and spatial experience rather than simply adding isolated natural sounds.

Visual design should similarly aim for calibrated natural structure rather than maximum vegetation density or visual intricacy (Laumann et al., 2001; Han, 2003; Hagerhall et al., 2004; Stamps, 2004; Li and Sullivan, 2016; Stoltz et al., 2016; Stevens, 2018; Lai et al., 2020; Liu et al., 2021; Robles et al., 2021). Layered canopy–shrub–groundcover planting, coherent blue-green composition, organised vegetation edges, and clear sightlines may support visual engagement while maintaining spatial legibility. However, NEFD should not be used as a standalone planting standard. Spaces with similar NEFD values may differ substantially in vegetation coverage, water presence, openness, enclosure, visibility, and maintenance quality. Visual complexity should therefore be assessed together with other spatial attributes and the corresponding soundscape condition.

The NEFD–GSI framework may provide an exploratory route from environmental sensing to design evaluation and green-space management (Li and Sullivan, 2016; Stoltz et al., 2016; Dzhambov et al., 2020; Lai et al., 2020; Korpilo et al., 2024). Recent ecological-restoration research similarly highlights the value of spatially explicit assessment for identifying differentiated intervention priorities under heterogeneous environmental conditions (Wang X. et al., 2026; Wang Y. et al., 2026). First, frequently used campus spaces—including courtyards, shaded walkways, building entrances, library edges, dormitory green spaces, waterside paths, and outdoor study areas—can be documented using panoramic images and short sound recordings collected under comparable environmental conditions. Second, NEFD and GSI can be calculated and mapped to characterise the visual–acoustic profile of frequently used spaces. Third, these profiles may help identify candidate areas for further field evaluation and possible intervention. In low-GSI spaces, possible management responses could include noise-source control, service-route adjustment, acoustic buffering, or protection of natural sound sources. Low-NEFD spaces with acceptable acoustic conditions may warrant evaluation of whether richer but coherent natural-element structure would be beneficial, whereas high-NEFD spaces should not automatically receive additional planting and instead require consideration of visual legibility, enclosure, safety, and sightlines. Spaces showing a similar high-GSI and intermediate-NEFD profile may be considered candidates for further field-based evaluation before major intervention. Finally, repeated image and audio collection after intervention can support post-occupancy evaluation and long-term management. Changes in NEFD and GSI can be compared with user perception, behavioural observation, and physiological or experience-sampling data to examine whether changes in the environmental profile correspond with changes in actual use and user response. In this way, the framework may provide supplementary information for campus renewal assessment, retrofit prioritisation, maintenance evaluation, and communication among designers, facility managers, and campus decision-makers.

### 4.5 Limitations and future research

Several limitations should be considered when interpreting the findings. First, each NEFD–GSI matrix cell was represented by one real campus scene. Although this strategy preserved ecological realism and ensured systematic coverage of the visual–acoustic parameter space, scene identity could not be separated completely from the corresponding NEFD–GSI combination. Vegetation coverage, water presence, openness, enclosure, maintenance quality, surrounding building form, and other unmeasured spatial attributes may have varied together with the focal indicators. The mixed-effects results should therefore be interpreted as response differences among selected structured scene conditions rather than as independent causal effects of NEFD or GSI. Similarly, the response-surface ranges were interpolated from nine representative conditions and should be treated as sample-specific design references rather than validated optimisation thresholds. The post-hoc sensitivity estimates further indicated greater sensitivity to moderate pupil-diameter effects than to electrodermal effects of comparable relative magnitude. Accordingly, the more selective SCL findings and other weak or null response patterns should be interpreted cautiously, particularly when true effects may fall below the detectable range of the present design. Future studies should include multiple independent scenes within each NEFD–GSI condition or use systematically controlled virtual environments to separate the focal indicators from other visual and spatial attributes. Accordingly, statistical significance in the participant-level models should not be interpreted as establishing generalisable scene-level effects attributable specifically to NEFD or GSI.

Second, the ecological validity of the VR protocol is necessarily limited by the contrast between stationary exposure to 360° panoramas and the dynamic way campus landscapes are experienced in everyday life (Browning et al., 2019; Knaust et al., 2022; Kawai et al., 2024; Kawai et al., 2025; Sella et al., 2025). Participants remained seated during relatively short, controlled exposures, whereas real campus use typically involves walking, head and body movement, route choice, changing viewing directions, transitions between enclosed and open spaces, and continuous shifts in visual and acoustic information. These behaviours can alter visual attention, optic flow, sound-source localisation, perceived enclosure, and physiological activation. Real-world campus experience is also socially embedded: pedestrians, conversations, crowd density, perceived safety, and opportunities for interaction can modify both restorative appraisal and soundscape perception. In addition, outdoor exposure includes thermal and microclimatic factors—including solar radiation, air temperature, humidity, wind, shade, and seasonal variation—that were absent from the present protocol but may interact with visual and acoustic conditions in shaping comfort and restoration. Therefore, the present findings should be interpreted as responses to controlled audiovisual representations of campus environments rather than direct estimates of restorative effects during actual campus use.

Future validation should consequently move beyond static seated VR exposure toward mobile and field-based protocols. Studies could combine ambulatory eye tracking, wearable EDA and cardiovascular monitoring, GPS-based movement trajectories, *in-situ* sound-level and microclimate measurements, and repeated experience-sampling assessments during walking, resting, and social use. Comparing the same locations across controlled VR, stationary field exposure, and active walking conditions would help determine which NEFD–GSI patterns are robust to changes in movement, social context, and thermal environment.

Third, generalisability is limited by the participant group and environmental context. Participants were university students, and all scenes were sampled from campuses in Suzhou, China. The multi-campus design captured variation across different campus forms within one urban-climatic context, but it does not establish city-wide, cross-cultural, or climate-independent thresholds (Liu F. et al., 2022; Liu Q. et al., 2022; Aghabozorgi et al., 2024; Huang et al., 2024; Menardo et al., 2024; Xu et al., 2024). Additional campuses, seasons, climates, cultural settings, and user groups are required to determine whether the high-GSI and medium-NEFD tendency remains stable or shifts with vegetation type, campus morphology, soundscape preference, and user characteristics (White et al., 2013; Markevych et al., 2017; Twohig-Bennett and Jones, 2018).

A key direction for future research is to determine whether the nonlinear patterns observed here can be reproduced as robust dose–response relationships. This will require denser sampling across the NEFD and GSI continua, multiple independent scenes at comparable exposure levels, and replication across different types of blue–green environments, including campuses, urban parks, streetscapes, waterfronts, and wetlands. Longitudinal and repeated-exposure designs are also needed to distinguish short-term response patterns from more stable restorative effects and to test for plateaus, turning points, or context-dependent critical ranges. Such work would help determine whether quantitative exposure ranges can eventually support more precise environmental-health and landscape-planning interventions. Until such validation is available, however, the numerical ranges observed in the present study should remain sample-specific empirical references rather than transferable design thresholds.

Future research should also combine controlled VR experiments with field-based post-occupancy evaluation (Li and Sullivan, 2016; Stoltz et al., 2016; Dzhambov et al., 2020; Lai et al., 2020; Korpilo et al., 2024). Linking NEFD and GSI with actual space use, behavioural observation, short experience-sampling surveys, and long-term environmental monitoring would provide stronger evidence for campus renewal and sustainable built-environment management.

## 5 Conclusion

This study developed a VR-based visual–acoustic assessment approach to examine how GSI-based natural sound-source dominance and natural-element visual complexity were associated with short-term psychophysiological responses in campus green infrastructure. NEFD was used to quantify the contour-based structural complexity of segmented natural elements, while GSI represented the relative dominance of natural sound components. Based on 73 audiovisual scenes collected from 10 campuses in Suzhou, nine representative NEFD–GSI conditions were selected for repeated-measures visual-only and audiovisual VR exposure. Subjective evaluation, eye-tracking, electrodermal, and cardiovascular indicators were integrated to examine response differences across the sampled visual–acoustic conditions.

Within the nine sampled Suzhou campus scene conditions, response differences were more consistently aligned with the GSI dimension. Higher-GSI scene conditions were generally accompanied by smaller stress-referenced pupil changes, greater electrodermal reduction, and more favourable subjective evaluations. NEFD-related response differences were non-monotonic and context-dependent across the sampled scene conditions. These patterns should not be interpreted as generalisable effects attributable independently to GSI or NEFD because each NEFD–GSI combination was represented by a single real scene. Instead, the observed NEFD-related pattern was non-monotonic and context-dependent, with medium-NEFD conditions showing favourable tendencies for several outcomes under medium- and high-GSI conditions. The comparison between visual-only and audiovisual exposure further showed that matched acoustic information did not produce a uniform overall improvement; its associated response differences varied across NEFD–GSI combinations and response systems. These findings support the broader principle that campus restorative performance depends on the coordination of visual natural structure and acoustic context rather than on maximising visible greenness or natural-element complexity alone.

Eye-tracking indicators showed the clearest differentiation across the short-duration visual–acoustic conditions, whereas electrodermal responses showed more selective associations and most cardiovascular indicators showed limited differentiation. These findings suggest that different response systems may operate over different temporal scales in short-duration VR exposure. Cardiovascular responses, in particular, may require longer exposure periods, repeated observations, or field-based monitoring before robust environmental differences can be established.

The study contributes to campus green-space research by integrating image-derived natural-element structure and sound-source-derived acoustic naturalness within a common NEFD–GSI representation and linking this representation with multisystem human-response measures. The framework may provide an exploratory basis for characterising visual–acoustic environmental profiles and identifying candidate spaces for further field evaluation. Panoramic images and field recordings may also support repeated assessment before and after intervention when interpreted together with user perception, behaviour, and other environmental characteristics. These applications should, however, be regarded as exploratory rather than as a validated optimisation or decision-support model.

The findings remain bounded by the present research design. Each NEFD–GSI matrix cell was represented by one real campus scene, so the focal indicators could not be separated completely from other scene characteristics such as vegetation coverage, water presence, openness, enclosure, maintenance quality, and surrounding built form. The exploratory response-surface analysis identified an empirical region of approximately GSI = 0.71–1.00 and NEFD = 1.31–1.52 within the present sample. These numerical ranges were derived from only nine representative Suzhou campus scenes and should be interpreted solely as sample- and context-specific empirical references, not as universal landscape-design thresholds, optimal values, or transferable design standards. Because the experiment used short-duration, seated exposure to static 360° VR panoramas, further field validation is required to determine whether these patterns remain robust during real-world walking, social interaction, and changing microclimatic conditions. Therefore, the proposed NEFD–GSI framework should currently be understood as an exploratory assessment logic for generating field-validation hypotheses, rather than as direct evidence of real-world landscape performance. Future research should use denser sampling and multiple independent scenes across comparable NEFD–GSI conditions, extend validation to diverse blue–green space types and populations, and employ longitudinal or repeated-exposure designs to test whether reproducible plateaus, turning points, or dose–response ranges can ultimately be established.

## Data Availability

The raw data supporting the conclusions of this article will be made available by the authors, without undue reservation.
