# Supplementary material for: Associations of soundscape naturalness and natural-element complexity with short-term psychophysiological restoration in campus green spaces: a virtual reality study
Source: Front Psychol. 2026 Sep 23;17:1947314. doi: 10.3389/fpsyg.2026.1947314 (PMC13642223; doi:10.3389/fpsyg.2026.1947314)
Supplement: Supplementary file 1 [file Supplementary_file_1.docx]

**Supplementary Table S1.** Demographic characteristics of the initially recruited and final analytical samples.

| **Characteristic** | **Recruited sample (n = 40)** | **Final sample (n = 35)** |
| --- | --- | --- |
| Age, years, mean ± SD | 22.1 ± 2.3 | 21.9 ± 2.2 |
| Age range, years | 18–28 | 18–28 |
| Female, n (%) | 20 (50.0%) | 17 (48.6%) |
| Male, n (%) | 20 (50.0%) | 18 (51.4%) |
| Engineering, n (%) | 18 (45.0%) | 17 (48.6%) |
| Natural sciences, n (%) | 10 (25.0%) | 8 (22.9%) |
| Humanities, n (%) | 12 (30.0%) | 10 (28.6%) |
| Prior VR experience, n (%) | 7 (17.5%) | 7 (20.0%) |

**Supplementary Table S2.** Image-based luminance characteristics of the nine luminance-matched VR panoramic stimuli.

| **Scene ID** | **Mean RGB intensity** | **Spherical-area-weighted linear relative luminance** |
| --- | --- | --- |
| B08 | 142.681 | 0.394312 |
| C02 | 141.895 | 0.393007 |
| D08 | 157.453 | 0.395291 |
| F01 | 140.659 | 0.393797 |
| F02 | 132.684 | 0.39432 |
| F03 | 162.978 | 0.39525 |
| F05 | 141.412 | 0.394831 |
| I05 | 157.025 | 0.395293 |
| I07 | 156.202 | 0.395323 |

**Supplementary Table S3.** Complete fixed-effect results from linear mixed-effects models of psychophysiological responses.

| **Domain** | **Outcome** | **Fixed effect** | **F(df1, df2)** | **exact p** | **FDR-adjusted q** | **partial η²** |
| --- | --- | --- | --- | --- | --- | --- |
| Cognitive | Pupil diameter | Condition | F(1, 578) = 0.173 | 0.678 | 0.814 | 0.0003 |
| Cognitive | Pupil diameter | NEFD | F(2, 578) = 15.028 | 4.34 × 10^-7^ | 2.43 × 10^-6^ | 0.0494 |
| Cognitive | Pupil diameter | GSI | F(2, 578) = 241.150 | 7.05 × 10^-77^ | 1.97 × 10^-75^ | 0.4549 |
| Cognitive | Pupil diameter | Condition × NEFD | F(2, 578) = 1.437 | 0.238 | 0.445 | 0.0049 |
| Cognitive | Pupil diameter | Condition × GSI | F(2, 578) = 0.507 | 0.602 | 0.814 | 0.0018 |
| Cognitive | Pupil diameter | NEFD × GSI | F(4, 578) = 7.411 | 7.97 × 10^-6^ | 2.79 × 10^-5^ | 0.0488 |
| Cognitive | Pupil diameter | Condition × NEFD × GSI | F(4, 578) = 0.891 | 0.469 | 0.729 | 0.0061 |
| Cognitive | Saccade frequency | Condition | F(1, 578) = 0.000 | 0.998 | 0.998 | 0.0000 |
| Cognitive | Saccade frequency | NEFD | F(2, 578) = 6.954 | 0.001 | 0.003 | 0.0235 |
| Cognitive | Saccade frequency | GSI | F(2, 578) = 81.308 | 7.67 × 10^-32^ | 5.37 × 10^-31^ | 0.2196 |
| Cognitive | Saccade frequency | Condition × NEFD | F(2, 578) = 0.289 | 0.749 | 0.839 | 0.0010 |
| Cognitive | Saccade frequency | Condition × GSI | F(2, 578) = 0.044 | 0.957 | 0.992 | 0.0002 |
| Cognitive | Saccade frequency | NEFD × GSI | F(4, 578) = 3.472 | 0.008 | 0.019 | 0.0235 |
| Cognitive | Saccade frequency | Condition × NEFD × GSI | F(4, 578) = 0.397 | 0.811 | 0.874 | 0.0027 |
| Cognitive | Saccade count | Condition | F(1, 578) = 0.822 | 0.365 | 0.601 | 0.0014 |
| Cognitive | Saccade count | NEFD | F(2, 578) = 12.890 | 3.33 × 10^-6^ | 1.33 × 10^-5^ | 0.0427 |
| Cognitive | Saccade count | GSI | F(2, 578) = 145.991 | 4.77 × 10^-52^ | 4.45 × 10^-51^ | 0.3356 |
| Cognitive | Saccade count | Condition × NEFD | F(2, 578) = 0.429 | 0.651 | 0.814 | 0.0015 |
| Cognitive | Saccade count | Condition × GSI | F(2, 578) = 1.767 | 0.172 | 0.344 | 0.0061 |
| Cognitive | Saccade count | NEFD × GSI | F(4, 578) = 3.889 | 0.004 | 0.010 | 0.0262 |
| Cognitive | Saccade count | Condition × NEFD × GSI | F(4, 578) = 0.590 | 0.67 | 0.814 | 0.0041 |
| Cognitive | Fixation count | Condition | F(1, 578) = 0.884 | 0.348 | 0.601 | 0.0015 |
| Cognitive | Fixation count | NEFD | F(2, 578) = 13.115 | 2.69 × 10^-6^ | 1.26 × 10^-5^ | 0.0434 |
| Cognitive | Fixation count | GSI | F(2, 578) = 147.998 | 1.26 × 10^-52^ | 1.77 × 10^-51^ | 0.3387 |
| Cognitive | Fixation count | Condition × NEFD | F(2, 578) = 0.448 | 0.639 | 0.814 | 0.0015 |
| Cognitive | Fixation count | Condition × GSI | F(2, 578) = 1.783 | 0.169 | 0.344 | 0.0061 |
| Cognitive | Fixation count | NEFD × GSI | F(4, 578) = 3.944 | 0.004 | 0.010 | 0.0266 |
| Cognitive | Fixation count | Condition × NEFD × GSI | F(4, 578) = 0.552 | 0.697 | 0.814 | 0.0038 |
| EDA | SCL | Condition | F(1, 578) = 0.006 | 0.938 | 0.938 | 0.0000 |
| EDA | SCL | NEFD | F(2, 578) = 2.226 | 0.109 | 0.286 | 0.0076 |
| EDA | SCL | GSI | F(2, 578) = 14.854 | 5.12 × 10^-7^ | 1.08 × 10^-5^ | 0.0489 |
| EDA | SCL | Condition × NEFD | F(2, 578) = 0.211 | 0.810 | 0.938 | 0.0007 |
| EDA | SCL | Condition × GSI | F(2, 578) = 0.279 | 0.757 | 0.938 | 0.0010 |
| EDA | SCL | NEFD × GSI | F(4, 578) = 2.652 | 0.032 | 0.157 | 0.0180 |
| EDA | SCL | Condition × NEFD × GSI | F(4, 578) = 8.549 | 1.05 × 10^-6^ | 1.10 × 10^-5^ | 0.0559 |
| EDA | SCR count | Condition | F(1, 578) = 0.147 | 0.701 | 0.938 | 0.0003 |
| EDA | SCR count | NEFD | F(2, 578) = 1.081 | 0.340 | 0.649 | 0.0037 |
| EDA | SCR count | GSI | F(2, 578) = 2.966 | 0.052 | 0.157 | 0.0102 |
| EDA | SCR count | Condition × NEFD | F(2, 578) = 0.307 | 0.736 | 0.938 | 0.0011 |
| EDA | SCR count | Condition × GSI | F(2, 578) = 0.516 | 0.597 | 0.938 | 0.0018 |
| EDA | SCR count | NEFD × GSI | F(4, 578) = 5.563 | 2.12 × 10^-4^ | 0.001 | 0.0371 |
| EDA | SCR count | Condition × NEFD × GSI | F(4, 578) = 2.427 | 0.047 | 0.157 | 0.0165 |
| EDA | SCR amplitude | Condition | F(1, 578) = 0.025 | 0.874 | 0.938 | 0.0000 |
| EDA | SCR amplitude | NEFD | F(2, 578) = 0.082 | 0.922 | 0.938 | 0.0003 |
| EDA | SCR amplitude | GSI | F(2, 578) = 1.259 | 0.285 | 0.598 | 0.0043 |
| EDA | SCR amplitude | Condition × NEFD | F(2, 578) = 0.224 | 0.799 | 0.938 | 0.0008 |
| EDA | SCR amplitude | Condition × GSI | F(2, 578) = 0.481 | 0.619 | 0.938 | 0.0017 |
| EDA | SCR amplitude | NEFD × GSI | F(4, 578) = 2.452 | 0.045 | 0.157 | 0.0167 |
| EDA | SCR amplitude | Condition × NEFD × GSI | F(4, 578) = 1.409 | 0.229 | 0.535 | 0.0097 |
| PPG/HRV | HR | Condition | F(1, 578) = 0.259 | 0.611 | 0.952 | 0.0004 |
| PPG/HRV | HR | NEFD | F(2, 578) = 1.142 | 0.320 | 0.952 | 0.0039 |
| PPG/HRV | HR | GSI | F(2, 578) = 1.448 | 0.236 | 0.943 | 0.0050 |
| PPG/HRV | HR | Condition × NEFD | F(2, 578) = 0.674 | 0.510 | 0.952 | 0.0023 |
| PPG/HRV | HR | Condition × GSI | F(2, 578) = 0.269 | 0.764 | 0.952 | 0.0009 |
| PPG/HRV | HR | NEFD × GSI | F(4, 578) = 0.902 | 0.463 | 0.952 | 0.0062 |
| PPG/HRV | HR | Condition × NEFD × GSI | F(4, 578) = 0.970 | 0.423 | 0.952 | 0.0067 |
| PPG/HRV | SDNN | Condition | F(1, 578) = 0.353 | 0.553 | 0.952 | 0.0006 |
| PPG/HRV | SDNN | NEFD | F(2, 578) = 0.287 | 0.751 | 0.952 | 0.0010 |
| PPG/HRV | SDNN | GSI | F(2, 578) = 0.776 | 0.461 | 0.952 | 0.0027 |
| PPG/HRV | SDNN | Condition × NEFD | F(2, 578) = 0.437 | 0.646 | 0.952 | 0.0015 |
| PPG/HRV | SDNN | Condition × GSI | F(2, 578) = 1.934 | 0.146 | 0.800 | 0.0066 |
| PPG/HRV | SDNN | NEFD × GSI | F(4, 578) = 0.756 | 0.554 | 0.952 | 0.0052 |
| PPG/HRV | SDNN | Condition × NEFD × GSI | F(4, 578) = 1.642 | 0.162 | 0.800 | 0.0112 |
| PPG/HRV | RMSSD | Condition | F(1, 578) = 0.021 | 0.884 | 0.952 | 0.0000 |
| PPG/HRV | RMSSD | NEFD | F(2, 578) = 0.187 | 0.829 | 0.952 | 0.0006 |
| PPG/HRV | RMSSD | GSI | F(2, 578) = 0.267 | 0.766 | 0.952 | 0.0009 |
| PPG/HRV | RMSSD | Condition × NEFD | F(2, 578) = 0.127 | 0.881 | 0.952 | 0.0004 |
| PPG/HRV | RMSSD | Condition × GSI | F(2, 578) = 1.769 | 0.171 | 0.800 | 0.0061 |
| PPG/HRV | RMSSD | NEFD × GSI | F(4, 578) = 0.501 | 0.735 | 0.952 | 0.0035 |
| PPG/HRV | RMSSD | Condition × NEFD × GSI | F(4, 578) = 2.157 | 0.073 | 0.677 | 0.0147 |
| PPG/HRV | LF/HF | Condition | F(1, 578) = 0.028 | 0.867 | 0.952 | 0.0000 |
| PPG/HRV | LF/HF | NEFD | F(2, 578) = 0.034 | 0.966 | 0.966 | 0.0001 |
| PPG/HRV | LF/HF | GSI | F(2, 578) = 0.201 | 0.818 | 0.952 | 0.0007 |
| PPG/HRV | LF/HF | Condition × NEFD | F(2, 578) = 0.083 | 0.920 | 0.954 | 0.0003 |
| PPG/HRV | LF/HF | Condition × GSI | F(2, 578) = 0.900 | 0.407 | 0.952 | 0.0031 |
| PPG/HRV | LF/HF | NEFD × GSI | F(4, 578) = 12.011 | 2.21 × 10^-9^ | 6.18 × 10^-8^ | 0.0767 |
| PPG/HRV | LF/HF | Condition × NEFD × GSI | F(4, 578) = 8.914 | 5.46 × 10^-7^ | 7.64 × 10^-6^ | 0.0581 |
|  |  |  |  |  |  |  |

**Supplementary Table S4.** Tukey-adjusted post hoc contrasts for FDR-supported interaction effects.

| **Outcome** | **Simple effect** | **Contrast** | **Estimate** | **95% CI** | **Tukey-adjusted p** | **EMM1 [95% CI]** | **EMM2 [95% CI]** | **Domain** | **Omnibus interaction** | **Source FDR q** |
| --- | --- | --- | --- | --- | --- | --- | --- | --- | --- | --- |
| Pupil diameter | NEFD within Low GSI | Low - Medium | 0.206 | [0.106, 0.306] | 5.01 × 10^-6^ | 2.289 [2.229, 2.350] | 2.084 [2.023, 2.145] | Cognitive | NEFD × GSI | 1.28 × 10^-4^ |
| Pupil diameter | NEFD within Low GSI | Low - High | 0.401 | [0.301, 0.501] | 5.06 × 10^-10^ | 2.289 [2.229, 2.350] | 1.889 [1.828, 1.949] | Cognitive | NEFD × GSI | 1.28 × 10^-4^ |
| Pupil diameter | NEFD within Low GSI | Medium - High | 0.195 | [0.095, 0.295] | 1.60 × 10^-5^ | 2.084 [2.023, 2.145] | 1.889 [1.828, 1.949] | Cognitive | NEFD × GSI | 1.28 × 10^-4^ |
| Pupil diameter | NEFD within Medium GSI | Low - Medium | 0.452 | [0.352, 0.552] | 5.06 × 10^-10^ | 1.619 [1.558, 1.680] | 1.167 [1.106, 1.228] | Cognitive | NEFD × GSI | 1.28 × 10^-4^ |
| Pupil diameter | NEFD within Medium GSI | Low - High | 0.286 | [0.186, 0.385] | 6.37 × 10^-10^ | 1.619 [1.558, 1.680] | 1.333 [1.272, 1.394] | Cognitive | NEFD × GSI | 1.28 × 10^-4^ |
| Pupil diameter | NEFD within Medium GSI | Medium - High | -0.166 | [-0.266, -0.067] | 2.96 × 10^-4^ | 1.167 [1.106, 1.228] | 1.333 [1.272, 1.394] | Cognitive | NEFD × GSI | 1.28 × 10^-4^ |
| Pupil diameter | NEFD within High GSI | Low - Medium | 0.49 | [0.390, 0.589] | 5.05 × 10^-10^ | 0.929 [0.868, 0.990] | 0.439 [0.378, 0.500] | Cognitive | NEFD × GSI | 1.28 × 10^-4^ |
| Pupil diameter | NEFD within High GSI | Low - High | 0.252 | [0.152, 0.352] | 1.57 × 10^-8^ | 0.929 [0.868, 0.990] | 0.677 [0.616, 0.737] | Cognitive | NEFD × GSI | 1.28 × 10^-4^ |
| Pupil diameter | NEFD within High GSI | Medium - High | -0.237 | [-0.337, -0.137] | 1.10 × 10^-7^ | 0.439 [0.378, 0.500] | 0.677 [0.616, 0.737] | Cognitive | NEFD × GSI | 1.28 × 10^-4^ |
| Saccade frequency | NEFD within Low GSI | Low - Medium | 3.014 | [0.508, 5.519] | 0.014 | 37.103 [35.622, 38.584] | 34.089 [32.608, 35.570] | Cognitive | NEFD × GSI | 0.033 |
| Saccade frequency | NEFD within Low GSI | Low - High | 6.094 | [3.589, 8.600] | 5.32 × 10^-8^ | 37.103 [35.622, 38.584] | 31.009 [29.528, 32.490] | Cognitive | NEFD × GSI | 0.033 |
| Saccade frequency | NEFD within Low GSI | Medium - High | 3.081 | [0.575, 5.587] | 0.011 | 34.089 [32.608, 35.570] | 31.009 [29.528, 32.490] | Cognitive | NEFD × GSI | 0.033 |
| Saccade frequency | NEFD within Medium GSI | Low - Medium | 6.386 | [3.880, 8.892] | 1.17 × 10^-8^ | 27.640 [26.159, 29.121] | 21.254 [19.773, 22.735] | Cognitive | NEFD × GSI | 0.033 |
| Saccade frequency | NEFD within Medium GSI | Low - High | 4.879 | [2.374, 7.385] | 1.73 × 10^-5^ | 27.640 [26.159, 29.121] | 22.760 [21.279, 24.241] | Cognitive | NEFD × GSI | 0.033 |
| Saccade frequency | NEFD within High GSI | Low - Medium | 7.082 | [4.576, 9.587] | 7.21 × 10^-10^ | 17.608 [16.127, 19.089] | 10.527 [9.046, 12.008] | Cognitive | NEFD × GSI | 0.033 |
| Saccade frequency | NEFD within High GSI | Low - High | 3.246 | [0.740, 5.752] | 0.007 | 17.608 [16.127, 19.089] | 14.362 [12.881, 15.843] | Cognitive | NEFD × GSI | 0.033 |
| Saccade frequency | NEFD within High GSI | Medium - High | -3.836 | [-6.342, -1.330] | 0.001 | 10.527 [9.046, 12.008] | 14.362 [12.881, 15.843] | Cognitive | NEFD × GSI | 0.033 |
| Saccade count | NEFD within Low GSI | Low - Medium | 8.882 | [3.815, 13.950] | 1.29 × 10^-4^ | 103.382 [100.307, 106.457] | 94.500 [91.425, 97.575] | Cognitive | NEFD × GSI | 0.021 |
| Saccade count | NEFD within Low GSI | Low - High | 13.282 | [8.215, 18.350] | 4.62 × 10^-9^ | 103.382 [100.307, 106.457] | 90.100 [87.025, 93.175] | Cognitive | NEFD × GSI | 0.021 |
| Saccade count | NEFD within Medium GSI | Low - Medium | 17.643 | [12.575, 22.710] | 5.06 × 10^-10^ | 78.871 [75.796, 81.946] | 61.229 [58.154, 64.304] | Cognitive | NEFD × GSI | 0.021 |
| Saccade count | NEFD within Medium GSI | Low - High | 7.786 | [2.718, 12.853] | 9.68 × 10^-4^ | 78.871 [75.796, 81.946] | 71.086 [68.011, 74.161] | Cognitive | NEFD × GSI | 0.021 |
| Saccade count | NEFD within Medium GSI | Medium - High | -9.857 | [-14.925, -4.790] | 1.77 × 10^-5^ | 61.229 [58.154, 64.304] | 71.086 [68.011, 74.161] | Cognitive | NEFD × GSI | 0.021 |
| Saccade count | NEFD within High GSI | Low - Medium | 16.2 | [11.133, 21.267] | 5.06 × 10^-10^ | 54.557 [51.482, 57.632] | 38.357 [35.282, 41.432] | Cognitive | NEFD × GSI | 0.021 |
| Saccade count | NEFD within High GSI | Medium - High | -11.278 | [-16.345, -6.210] | 7.12 × 10^-7^ | 38.357 [35.282, 41.432] | 49.635 [46.560, 52.710] | Cognitive | NEFD × GSI | 0.021 |
| Fixation count | NEFD within Low GSI | Low - Medium | 9.795 | [4.260, 15.330] | 1.09 × 10^-4^ | 112.052 [108.691, 115.412] | 102.257 [98.897, 105.618] | Cognitive | NEFD × GSI | 0.021 |
| Fixation count | NEFD within Low GSI | Low - High | 14.623 | [9.088, 20.158] | 3.58 × 10^-9^ | 112.052 [108.691, 115.412] | 97.429 [94.068, 100.789] | Cognitive | NEFD × GSI | 0.021 |
| Fixation count | NEFD within Medium GSI | Low - Medium | 19.543 | [14.008, 25.078] | 5.06 × 10^-10^ | 85.129 [81.768, 88.489] | 65.586 [62.225, 68.946] | Cognitive | NEFD × GSI | 0.021 |
| Fixation count | NEFD within Medium GSI | Low - High | 8.614 | [3.079, 14.149] | 8.13 × 10^-4^ | 85.129 [81.768, 88.489] | 76.514 [73.154, 79.875] | Cognitive | NEFD × GSI | 0.021 |
| Fixation count | NEFD within Medium GSI | Medium - High | -10.929 | [-16.464, -5.394] | 1.29 × 10^-5^ | 65.586 [62.225, 68.946] | 76.514 [73.154, 79.875] | Cognitive | NEFD × GSI | 0.021 |
| Fixation count | NEFD within High GSI | Low - Medium | 17.786 | [12.251, 23.321] | 5.06 × 10^-10^ | 58.357 [54.997, 61.718] | 40.571 [37.211, 43.932] | Cognitive | NEFD × GSI | 0.021 |
| Fixation count | NEFD within High GSI | Medium - High | -12.313 | [-17.848, -6.778] | 7.22 × 10^-7^ | 40.571 [37.211, 43.932] | 52.884 [49.523, 56.245] | Cognitive | NEFD × GSI | 0.021 |
| SCL | Condition within Medium NEFD × High GSI | Visual - Audiovisual | 6.626 | [5.038, 8.215] | 1.65 × 10^-15^ | -3.098 [-4.378, -1.817] | -9.724 [-11.005, -8.443] | EDA | Condition × NEFD × GSI | 1.26 × 10^-5^ |
| SCR count | NEFD within High GSI | Low - Medium | 7.043 | [4.112, 9.973] | 7.73 × 10^-8^ | -4.100 [-5.920, -2.280] | -11.143 [-12.962, -9.323] | EDA | NEFD × GSI | 0.001 |
| SCR count | NEFD within High GSI | Low - High | 5.316 | [2.385, 8.246] | 7.01 × 10^-5^ | -4.100 [-5.920, -2.280] | -9.416 [-11.235, -7.596] | EDA | NEFD × GSI | 0.001 |
| LF/HF | NEFD within High GSI | Low - Medium | 2.274 | [0.888, 3.661] | 3.80 × 10^-4^ | -1.007 [-1.867, -0.147] | -3.281 [-4.141, -2.421] | PPG/HRV | NEFD × GSI | 3.53 × 10^-8^ |
| LF/HF | NEFD within High GSI | Medium - High | -2.743 | [-4.130, -1.356] | 1.24 × 10^-5^ | -3.281 [-4.141, -2.421] | -0.538 [-1.398, 0.322] | PPG/HRV | NEFD × GSI | 3.53 × 10^-8^ |
| LF/HF | Condition within Low NEFD × High GSI | Visual - Audiovisual | 1.665 | [0.026, 3.305] | 0.046 | -0.174 [-1.362, 1.014] | -1.839 [-3.027, -0.652] | PPG/HRV | Condition × NEFD × GSI | 4.37 × 10^-6^ |
| LF/HF | Condition within Medium NEFD × High GSI | Visual - Audiovisual | -6.157 | [-7.796, -4.518] | 5.65 × 10^-13^ | -6.360 [-7.547, -5.172] | -0.202 [-1.390, 0.985] | PPG/HRV | Condition × NEFD × GSI | 4.37 × 10^-6^ |

**Supplementary Table S5.** Complete fixed-effect results from linear mixed-effects models of subjective responses.

| **Domain** | **Outcome** | **Fixed effect** | **F(df1, df2)** | **exact p** | **FDR-adjusted q** | **partial η²** |
| --- | --- | --- | --- | --- | --- | --- |
| PRS | Being away | Condition | F(1, 578) = 453.140 | 1.11 × 10^-74^ | 4.61 × 10^-74^ | 0.4395 |
| PRS | Being away | NEFD | F(2, 578) = 0.419 | 0.658 | 0.830 | 0.0014 |
| PRS | Being away | GSI | F(2, 578) = 1.411 | 0.245 | 0.407 | 0.0049 |
| PRS | Being away | Condition × NEFD | F(2, 578) = 4.397 | 0.013 | 0.032 | 0.0150 |
| PRS | Being away | Condition × GSI | F(2, 578) = 6.240 | 0.002 | 0.006 | 0.0211 |
| PRS | Being away | NEFD × GSI | F(4, 578) = 0.562 | 0.690 | 0.837 | 0.0039 |
| PRS | Being away | Condition × NEFD × GSI | F(4, 578) = 2.053 | 0.086 | 0.173 | 0.0140 |
| PRS | Fascination | Condition | F(1, 578) = 275.803 | 6.30 × 10^-51^ | 2.12 × 10^-50^ | 0.3230 |
| PRS | Fascination | NEFD | F(2, 578) = 742.899 | 1.81 × 10^-160^ | 1.27 × 10^-159^ | 0.7199 |
| PRS | Fascination | GSI | F(2, 578) = 2303.336 | 4.39 × 10^-276^ | 8.24 × 10^-275^ | 0.8885 |
| PRS | Fascination | Condition × NEFD | F(2, 578) = 23.771 | 1.20 × 10^-10^ | 3.76 × 10^-10^ | 0.0760 |
| PRS | Fascination | Condition × GSI | F(2, 578) = 253.135 | 1.10 × 10^-79^ | 4.78 × 10^-79^ | 0.4669 |
| PRS | Fascination | NEFD × GSI | F(4, 578) = 216.108 | 2.88 × 10^-113^ | 1.54 × 10^-112^ | 0.5993 |
| PRS | Fascination | Condition × NEFD × GSI | F(4, 578) = 10.705 | 2.24 × 10^-8^ | 6.80 × 10^-8^ | 0.0690 |
| PRS | Compatibility | Condition | F(1, 578) = 381.116 | 1.43 × 10^-65^ | 5.44 × 10^-65^ | 0.3974 |
| PRS | Compatibility | NEFD | F(2, 578) = 1365.352 | 1.04 × 10^-219^ | 1.35 × 10^-218^ | 0.8253 |
| PRS | Compatibility | GSI | F(2, 578) = 2303.060 | 4.53 × 10^-276^ | 8.24 × 10^-275^ | 0.8885 |
| PRS | Compatibility | Condition × NEFD | F(2, 578) = 225.991 | 3.09 × 10^-73^ | 1.22 × 10^-72^ | 0.4388 |
| PRS | Compatibility | Condition × GSI | F(2, 578) = 379.898 | 4.68 × 10^-106^ | 2.37 × 10^-105^ | 0.5679 |
| PRS | Compatibility | NEFD × GSI | F(4, 578) = 455.117 | 5.49 × 10^-177^ | 4.99 × 10^-176^ | 0.7590 |
| PRS | Compatibility | Condition × NEFD × GSI | F(4, 578) = 80.023 | 5.06 × 10^-54^ | 1.77 × 10^-53^ | 0.3564 |
| PRS | Extent | Condition | F(1, 578) = 377.921 | 3.77 × 10^-65^ | 1.37 × 10^-64^ | 0.3953 |
| PRS | Extent | NEFD | F(2, 578) = 680.744 | 1.14 × 10^-152^ | 7.39 × 10^-152^ | 0.7020 |
| PRS | Extent | GSI | F(2, 578) = 2520.146 | 3.64 × 10^-286^ | 3.32 × 10^-284^ | 0.8971 |
| PRS | Extent | Condition × NEFD | F(2, 578) = 2.853 | 0.058 | 0.124 | 0.0098 |
| PRS | Extent | Condition × GSI | F(2, 578) = 271.479 | 7.34 × 10^-84^ | 3.34 × 10^-83^ | 0.4844 |
| PRS | Extent | NEFD × GSI | F(4, 578) = 183.406 | 2.30 × 10^-101^ | 1.10 × 10^-100^ | 0.5593 |
| PRS | Extent | Condition × NEFD × GSI | F(4, 578) = 25.867 | 9.63 × 10^-20^ | 3.13 × 10^-19^ | 0.1518 |
| SAM | Valence | Condition | F(1, 578) = 1.349 | 0.246 | 0.407 | 0.0023 |
| SAM | Valence | NEFD | F(2, 578) = 1.261 | 0.284 | 0.443 | 0.0043 |
| SAM | Valence | GSI | F(2, 578) = 762.714 | 7.43 × 10^-163^ | 6.15 × 10^-162^ | 0.7252 |
| SAM | Valence | Condition × NEFD | F(2, 578) = 1.976 | 0.140 | 0.259 | 0.0068 |
| SAM | Valence | Condition × GSI | F(2, 578) = 0.252 | 0.777 | 0.881 | 0.0009 |
| SAM | Valence | NEFD × GSI | F(4, 578) = 0.318 | 0.866 | 0.916 | 0.0022 |
| SAM | Valence | Condition × NEFD × GSI | F(4, 578) = 1.028 | 0.392 | 0.557 | 0.0071 |
| SAM | Arousal | Condition | F(1, 578) = 0.011 | 0.918 | 0.940 | 0.0000 |
| SAM | Arousal | NEFD | F(2, 578) = 0.248 | 0.780 | 0.881 | 0.0009 |
| SAM | Arousal | GSI | F(2, 578) = 567.463 | 4.43 × 10^-137^ | 2.69 × 10^-136^ | 0.6626 |
| SAM | Arousal | Condition × NEFD | F(2, 578) = 0.212 | 0.809 | 0.887 | 0.0007 |
| SAM | Arousal | Condition × GSI | F(2, 578) = 0.231 | 0.793 | 0.881 | 0.0008 |
| SAM | Arousal | NEFD × GSI | F(4, 578) = 1.041 | 0.385 | 0.557 | 0.0072 |
| SAM | Arousal | Condition × NEFD × GSI | F(4, 578) = 0.495 | 0.740 | 0.874 | 0.0034 |
| SAM | Dominance | Condition | F(1, 578) = 2.199 | 0.139 | 0.259 | 0.0038 |
| SAM | Dominance | NEFD | F(2, 578) = 3.155 | 0.043 | 0.102 | 0.0108 |
| SAM | Dominance | GSI | F(2, 578) = 518.611 | 1.04 × 10^-129^ | 5.93 × 10^-129^ | 0.6422 |
| SAM | Dominance | Condition × NEFD | F(2, 578) = 4.046 | 0.018 | 0.044 | 0.0138 |
| SAM | Dominance | Condition × GSI | F(2, 578) = 0.778 | 0.460 | 0.644 | 0.0027 |
| SAM | Dominance | NEFD × GSI | F(4, 578) = 1.092 | 0.360 | 0.538 | 0.0075 |
| SAM | Dominance | Condition × NEFD × GSI | F(4, 578) = 1.941 | 0.102 | 0.202 | 0.0133 |
| NASA-TLX | Mental demand | Condition | F(1, 578) = 3.685 | 0.055 | 0.123 | 0.0063 |
| NASA-TLX | Mental demand | NEFD | F(2, 578) = 0.393 | 0.675 | 0.830 | 0.0014 |
| NASA-TLX | Mental demand | GSI | F(2, 578) = 1406.059 | 9.23 × 10^-223^ | 1.40 × 10^-221^ | 0.8295 |
| NASA-TLX | Mental demand | Condition × NEFD | F(2, 578) = 2.677 | 0.070 | 0.144 | 0.0092 |
| NASA-TLX | Mental demand | Condition × GSI | F(2, 578) = 0.648 | 0.524 | 0.717 | 0.0022 |
| NASA-TLX | Mental demand | NEFD × GSI | F(4, 578) = 0.594 | 0.667 | 0.830 | 0.0041 |
| NASA-TLX | Mental demand | Condition × NEFD × GSI | F(4, 578) = 1.587 | 0.176 | 0.314 | 0.0109 |
| NASA-TLX | Physical demand | Condition | F(1, 578) = 0.399 | 0.528 | 0.717 | 0.0007 |
| NASA-TLX | Physical demand | NEFD | F(2, 578) = 2.863 | 0.058 | 0.124 | 0.0098 |
| NASA-TLX | Physical demand | GSI | F(2, 578) = 744.294 | 1.23 × 10^-160^ | 9.30 × 10^-160^ | 0.7203 |
| NASA-TLX | Physical demand | Condition × NEFD | F(2, 578) = 1.598 | 0.203 | 0.356 | 0.0055 |
| NASA-TLX | Physical demand | Condition × GSI | F(2, 578) = 0.133 | 0.875 | 0.916 | 0.0005 |
| NASA-TLX | Physical demand | NEFD × GSI | F(4, 578) = 1.065 | 0.373 | 0.547 | 0.0073 |
| NASA-TLX | Physical demand | Condition × NEFD × GSI | F(4, 578) = 0.677 | 0.608 | 0.789 | 0.0047 |
| NASA-TLX | Temporal demand | Condition | F(1, 578) = 0.116 | 0.734 | 0.874 | 0.0002 |
| NASA-TLX | Temporal demand | NEFD | F(2, 578) = 4.704 | 0.009 | 0.024 | 0.0160 |
| NASA-TLX | Temporal demand | GSI | F(2, 578) = 1054.187 | 1.47 × 10^-193^ | 1.68 × 10^-192^ | 0.7848 |
| NASA-TLX | Temporal demand | Condition × NEFD | F(2, 578) = 0.154 | 0.857 | 0.916 | 0.0005 |
| NASA-TLX | Temporal demand | Condition × GSI | F(2, 578) = 0.525 | 0.592 | 0.789 | 0.0018 |
| NASA-TLX | Temporal demand | NEFD × GSI | F(4, 578) = 1.781 | 0.131 | 0.254 | 0.0122 |
| NASA-TLX | Temporal demand | Condition × NEFD × GSI | F(4, 578) = 0.319 | 0.865 | 0.916 | 0.0022 |
| NASA-TLX | Performance satisfaction | Condition | F(1, 578) = 0.253 | 0.615 | 0.789 | 0.0004 |
| NASA-TLX | Performance satisfaction | NEFD | F(2, 578) = 1.515 | 0.221 | 0.379 | 0.0052 |
| NASA-TLX | Performance satisfaction | GSI | F(2, 578) = 2306.668 | 3.03 × 10^-276^ | 8.24 × 10^-275^ | 0.8887 |
| NASA-TLX | Performance satisfaction | Condition × NEFD | F(2, 578) = 0.084 | 0.919 | 0.940 | 0.0003 |
| NASA-TLX | Performance satisfaction | Condition × GSI | F(2, 578) = 1.768 | 0.172 | 0.312 | 0.0061 |
| NASA-TLX | Performance satisfaction | NEFD × GSI | F(4, 578) = 3.424 | 0.009 | 0.024 | 0.0231 |
| NASA-TLX | Performance satisfaction | Condition × NEFD × GSI | F(4, 578) = 0.685 | 0.602 | 0.789 | 0.0047 |
| NASA-TLX | Effort | Condition | F(1, 578) = 0.000 | 1.000 | 1.000 | 0.0000 |
| NASA-TLX | Effort | NEFD | F(2, 578) = 5.713 | 0.003 | 0.010 | 0.0194 |
| NASA-TLX | Effort | GSI | F(2, 578) = 2332.735 | 1.69 × 10^-277^ | 7.68 × 10^-276^ | 0.8898 |
| NASA-TLX | Effort | Condition × NEFD | F(2, 578) = 0.238 | 0.788 | 0.881 | 0.0008 |
| NASA-TLX | Effort | Condition × GSI | F(2, 578) = 0.000 | 1.000 | 1.000 | 0.0000 |
| NASA-TLX | Effort | NEFD × GSI | F(4, 578) = 4.655 | 0.001 | 0.003 | 0.0312 |
| NASA-TLX | Effort | Condition × NEFD × GSI | F(4, 578) = 1.312 | 0.264 | 0.422 | 0.0090 |
| NASA-TLX | Frustration | Condition | F(1, 578) = 4.086 | 0.044 | 0.102 | 0.0070 |
| NASA-TLX | Frustration | NEFD | F(2, 578) = 2.951 | 0.053 | 0.121 | 0.0101 |
| NASA-TLX | Frustration | GSI | F(2, 578) = 1036.575 | 6.68 × 10^-192^ | 6.75 × 10^-191^ | 0.7820 |
| NASA-TLX | Frustration | Condition × NEFD | F(2, 578) = 1.362 | 0.257 | 0.418 | 0.0047 |
| NASA-TLX | Frustration | Condition × GSI | F(2, 578) = 1.022 | 0.361 | 0.538 | 0.0035 |
| NASA-TLX | Frustration | NEFD × GSI | F(4, 578) = 1.254 | 0.287 | 0.443 | 0.0086 |
| NASA-TLX | Frustration | Condition × NEFD × GSI | F(4, 578) = 0.430 | 0.787 | 0.881 | 0.0030 |
